# Dynamic physiological α-synuclein S129 phosphorylation is driven by neuronal activity

**DOI:** 10.1038/s41531-023-00444-w

**Published:** 2023-01-16

**Authors:** Nagendran Ramalingam, Shan-Xue Jin, Tim E. Moors, Luis Fonseca-Ornelas, Kazuma Shimanaka, Shi Lei, Hugh P. Cam, Aurelia Hays Watson, Lisa Brontesi, Lai Ding, Dinc Yasat Hacibaloglu, Haiyang Jiang, Se Joon Choi, Ellen Kanter, Lei Liu, Tim Bartels, Silke Nuber, David Sulzer, Eugene V. Mosharov, Weisheng V. Chen, Shaomin Li, Dennis J. Selkoe, Ulf Dettmer

**Affiliations:** 1 Ann Romney Center for Neurologic Diseases, Brigham and Women’s Hospital and Harvard Medical School, Boston, MA 02115 USA; 2 Leveragen, Inc., 17 Briden Street, Worcester, MA 01605 USA; 3 UK Dementia Research Institute, University College London, London, UK; 4 Division of Molecular Therapeutics, New York State Psychiatric Institute, Research Foundation for Mental Hygiene, New York, NY 10032 USA; 5 Departments of Neurology and Psychiatry, Columbia University Medical Center, New York, NY 10032 USA; 6 Department of Molecular Therapeutics and Pharmacology, Columbia University Medical Center, New York, NY 10032 USA

**Keywords:** Cell biology, Parkinson's disease

## Abstract

In Parkinson’s disease and other synucleinopathies, the elevation of α-synuclein phosphorylated at Serine129 (pS129) is a widely cited marker of pathology. However, the physiological role for pS129 has remained undefined. Here we use multiple approaches to show for the first time that pS129 functions as a physiological regulator of neuronal activity. Neuronal activity triggers a sustained increase of pS129 in cultured neurons (200% within 4 h). In accord, brain pS129 is elevated in environmentally enriched mice exhibiting enhanced long-term potentiation. Activity-dependent α-synuclein phosphorylation is S129-specific, reversible, confers no cytotoxicity, and accumulates at synapsin-containing presynaptic boutons. Mechanistically, our findings are consistent with a model in which neuronal stimulation enhances Plk2 kinase activity via a calcium/calcineurin pathway to counteract PP2A phosphatase activity for efficient phosphorylation of membrane-bound α-synuclein. Patch clamping of rat *SNCA*^*−/−*^ neurons expressing exogenous wild-type or phospho-incompetent (S129A) α-synuclein suggests that pS129 fine-tunes the balance between excitatory and inhibitory neuronal currents. Consistently, our novel S129A knock-in (S129A^KI^) mice exhibit impaired hippocampal plasticity. The discovery of a key physiological function for pS129 has implications for understanding the role of α-synuclein in neurotransmission and adds nuance to the interpretation of pS129 as a synucleinopathy biomarker.

## Introduction

Parkinson’s disease (PD), dementia with Lewy bodies (DLB), and multiple system atrophy (MSA) are collectively referred to as synucleinopathies. There are no approved therapies to slow or halt the debilitating progression of these diseases. The causative role of the neuronal protein α-synuclein (αS) is strongly linked to αS aggregates found in Lewy bodies/Lewy neurites (LBs/LNs), the pathological hallmarks of synucleinopathies. It has been estimated that 90% of aggregated αS present in LBs contains phospho-Serine129 (pS129)^1^, implying that this specific phosphorylation event is connected to pathology. However, researchers have posited disparate conclusions about the role of αS-pS129 (simply ‘pS129’ hereafter) in health and disease. Certain studies have reported that pS129 is present on monomeric and soluble αS and able to inhibit αS fibril formation under certain conditions^2^. Others have corroborated the initial observation of pS129 presence in LBs^1^ as evident by aggregated, oligomeric, and fibrillar pS129 in the human brain (e.g.,^3^). It has also been suggested that pS129 accelerates neuronal death in cell culture^4^ as well as in vivo^5^ and could potentially inhibit polo-like kinase 2 (Plk2) from properly regulating stress signaling^6^. However, others have proposed that pS129 does not confer toxicity in vivo^7–9^ and may actually be a neuroprotective mechanism accelerating the clearance of aggregated αS^10,11^. pS129 has also been suggested to be involved in αS turnover^11,12^ or in altering gene expression^13^.

The commonality of these studies is the widely cited conclusion that pS129 is associated with pathology. There are, however, sporadic reports that S129 phosphorylation may be a normal event in mouse brain^14^ or adult human brain^15^. Yet, the role, if any, for pS129 in normal neuronal function has not been addressed in these studies. Here, we report for the first time a physiological role for pS129 in fine-tuning neuronal function: pS129 arises normally in response to synaptic activity; activity-dependent pS129 is reversible and not associated with toxicity; other known phosphorylation sites are not altered; and activity-dependent pS129 is catalyzed by Plk2 downstream of calcineurin (CaN) and counteracted by protein phosphatase 2A (PP2A). We confirmed the functional relevance of pS129 by showing that αS with an S129A mutation (which prevents S129 phosphorylation) decreased the amplitude of spontaneous excitatory post-synaptic currents (sEPSCs) and increased spontaneous inhibitory post-synaptic currents (sIPSCs) in cultured neurons. Furthermore, our novel S129A^KI^ mice exhibited impaired paired-pulse facilitation, short-term plasticity, and LTP. Collectively, our unexpected findings are consistent with a feed-forward mechanism in which pS129 promotes neuronal activity by dampening an inhibitory role of αS on glutamatergic synaptic transmission. Lack of reversible pS129 may lead to neuronal hyperexcitability and accompanying pathology in PD.

## Results

### αS pS129 levels correlate with neuronal activity in vitro and in vivo

**Fig. 1:**
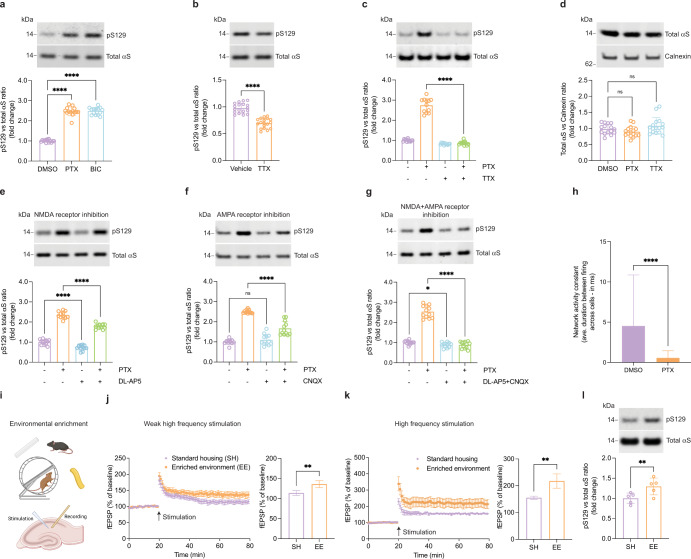
pS129 correlates with neuronal activity. **a** DIV17–21 rat cortical neurons treated with the GABA_A_ receptor antagonists picrotoxin (PTX, 20 µM) or bicuculline (BIC, 20 µM) for 2 h. WB for total αS and pS129. *N* = 3 independent experiments on different days, *n* = 14 biological replicates total. ****p < 0.0001. Mean +/− SD. Brown-Forsythe and Welch ANOVA with Dunnett’s T3 post hoc test for multiple comparisons. **b** DIV17-21 rat cortical neurons treated with vehicle or the sodium channel blocker tetrodotoxin (TTX, 1 µM). WB for total αS and pS129. *N* = 3 independent experiments on different days, *n* = 18 biological replicates total. ****p < 0.0001. Mean +/− SD. Unpaired *t*-tests with Welch’s correction; two-tailed. **c** DIV17-21 rat cortical neurons treated with PTX (20 µM), TTX (1 µM), or PTX + TTX. WB for total αS and pS129. *N* = 3 independent experiments on different days, *n* = 18 biological replicates total. ****p < 0.0001. Mean +/− SD. Brown-Forsythe and Welch ANOVA with Dunnett’s T3 post hoc test for multiple comparisons. **d** DIV17-21 rat cortical neurons treated with 20 µM PTX or 1 µM TTX for 2 h. WB for total αS and Calnexin (loading control). *N* = 2 independent experiments on different days, *n* = 16 biological replicates total. ns not significant. Mean +/− SD. Brown-Forsythe and Welch ANOVA with Dunnett’s T3 post hoc test for multiple comparisons. **e** DIV17-21 rat cortical neurons treated with 20 µM PTX, 25 µM DL-AP5 (NMDA receptor antagonist) or PTX + DL-AP5 for 2 h. WB for total αS and pS129. *N* = 3 independent experiments on different days, *n* = 12 biological replicates total; ***p < 0.001; ****p < 0.0001. Mean +/− SD. Brown-Forsythe and Welch ANOVA with Dunnett’s T3 post hoc test for multiple comparisons. **f** DIV17-21 rat cortical neurons treated with 20 µM PTX, 10 µM CNQX (AMPA receptor antagonist) or PTX + CNQX for 2 h. WB for total αS and pS129. *N* = 3 independent experiments on different days, *n* = 12 biological replicates total; ****p < 0.0001; ns not significant. Mean +/− SD. Brown-Forsythe and Welch ANOVA with Dunnett’s T3 post hoc test for multiple comparisons. **g** DIV17-21 rat cortical neurons treated with PTX, DL-AP5 + CNQX or PTX + DL-AP5 + CNQX for 2 h. WB for total αS and pS129. *N* = 3 independent experiments on different days, *n* = 12 biological replicates total; *p < 0.1; ****p < 0.0001; ns not significant. Mean +/− SD. Brown-Forsythe and Welch ANOVA with Dunnett’s T3 post hoc test for multiple comparisons. **h** Network activity constant of calcium transients, as expressed by inter-event interval between and across cells. *N* = 95 individual events for DMSO, *N* = 74 individual events for PTX. ****p < 0.0001. Mean +/− SD. Welch’s *t*-test; two-tailed. A total of 20 individual cells analyzed from one of the three independent experiments performed on different days. Time lapse movies related to this panel can be viewed in supplementary section—Supplementary Movie 2 (DMSO) and 3 (PTX). **i** Schematics of environmental enrichment and slice recording (created with BioRender.com). **j** LTP measurements in mouse hippocampal slices, weak high frequency stimulation. Mice kept under standard housing (SH) vs. enriched environment (EE). *N* = 5 hippocampal slices. **p < 0.002; Mean +/− SD. Unpaired *t*-test, two-tailed. **k** Analogous to **j**, but standard high-frequency stimulation (HFS). *N* = 5 hippocampal slices. **p < 0.002; Mean +/− SD. Unpaired *t*-test, two-tailed. **l** EE vs. SH mice: WB for total αS and pS129. *N* = 5 pairs of animals. **p < 0.002; Mean +/− SD. Paired *t*-test; two-tailed. A time lapse movie of mice training in the environmentally enriched cage can be viewed in supplementary section—Supplementary Movie 1.

To search for a possible effect of neuronal stimulation on pS129, we harvested embryonic rat cortical neurons and let them mature for ~3 weeks in culture. It has been shown that after 10 days in culture, αS is robustly expressed and becomes enriched at synapses^16^. We then tested a variety of stimuli for their ability to generate continuous neuronal activation, assessed by multi-electrode arrays and expression of c-fos. We observed robust effects by exposing cortical cultures to the GABA_A_-receptor antagonist picrotoxin (PTX; Supplementary Fig. 1a–c), which stimulates post-synaptic neurons by reducing inhibitory tone from GABAergic neurons in the culture. GABA_A_ blockers have been widely used to study activity-dependent processes relevant for physiological conditions^17–21^. We examined the effects of 2 h treatments with 20 µM PTX or bicuculline (BIC; another GABA_A_-receptor antagonist) on the levels of total αS and pS129 by Western blotting (WB). Both GABA_A_ receptor antagonists increased pS129 immunoreactivity by more than 100%. This was confirmed by two independent antibodies specific to pS129 (Fig. 1a and Supplementary Fig. 2a). The relative pS129 levels increased further over time and plateaued after ~6 h at approximately 350% of pre-treatment levels (Supplementary Fig. 2b). Next, we asked whether basal pS129 is also regulated by spontaneous synaptic activity in the culture. Treating unstimulated neurons for 2 h with 1 µM tetrodotoxin (TTX), a sodium channel blocker that prevents the propagation of action potentials^22^, reduced pS129 levels by about 25%, suggesting that a significant portion of basal pS129 is the result of spontaneous synaptic activity in our cultures (Fig. 1b). Moreover, PTX-induced pS129 was completely blocked by co-administering TTX (Fig. 1c). Total αS levels were unchanged by 2 h PTX or TTX treatment (Fig. 1d) and even after 24 h (Supplementary Fig. 3a–c), suggesting overall αS turnover is not altered by changes in neuronal activity. A lactate-dehydrogenase (LDH) release assay showed no evidence for cytotoxicity caused by 10, 20 or even 40 µM of PTX, in contrast to the positive control of 0.5% Triton X-100 (Supplementary Fig. 3d). This was not surprising because even 100 µM PTX or BIC treatment for 48 h have routinely been used to model homeostatic plasticity^18,23–25^. We concluded that continuous PTX stimulation promotes S129 phosphorylation without altering total αS levels or affecting the health of cultured cortical neurons. We next probed the involvement of glutamate receptors in activity-dependent pS129 elevation because the network activity of our rat cortical cultures is expected to depend largely on glutamatergic signaling. Indeed, we found PTX-induced pS129 to be significantly lessened by co-treatment with the NMDA receptor antagonist (2R)-amino-5-phosphonovaleric acid (DL-AP5; Fig. 1e) or the AMPA/kainate receptor antagonist cyanquixaline (CNQX; Fig. 1f). Interestingly, combined treatment with both antagonists fully blocked pS129 elevation (Fig. 1g). Calcium imaging (see “Methods” for details) confirmed that network activity, i.e., the synchronous firing of the cultured neurons, is strongly increased by PTX treatment (Fig. 1h and Supplementary Fig. 5a, b). The inter-event interval both within and across neurons as described by a network activity constant was almost 0 ms (highly synchronous) for PTX-stimulated neurons vs. ~5 ms for unstimulated neurons. Next, we sought to extend this observation to an in vivo setting and housed wild-type C57BL/6 mice (age 4 weeks) in enriched environment (EE) vs. standard housing (SH) for 8 weeks (Fig. 1i). As expected from prior work^26^, we found hippocampal slices of mice housed in EE to exhibit significantly enhanced Long Term Potentiation (LTP) upon stimulation with either weak (Fig. 1j) or standard high frequency (Fig. 1k). Strikingly, the EE mice exhibited an increase in pS129 levels relative to total αS (Fig. 1l), similar to our findings in PTX- or bicuculline-stimulated neurons in culture.

### Activity-dependent αS phosphorylation is S129-specific and reversible

**Fig. 2:**
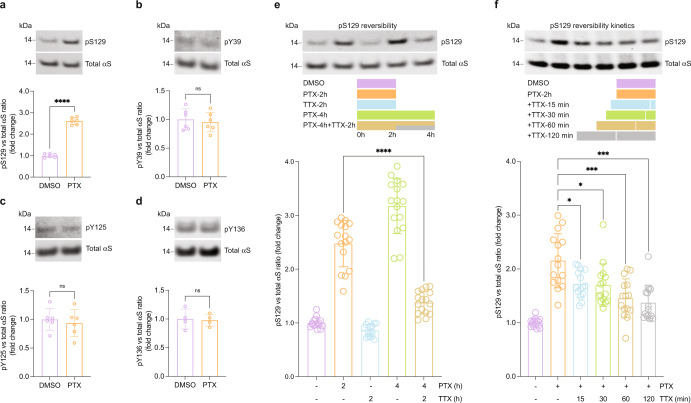
Activity-dependent αS phosphorylation is S129-specific and reversible. **a**–**d** DIV17-21 rat cortical neurons treated with 20 µM PTX for 2 h. WB for total αS and indicated phospho-sites. *N* = 6 (**a**–**c**) or 4 (**d**) independent experiments on different days. ****p < 0.0001; ns not significant. Mean +/− SD. Paired *t*-tests; two-tailed. **e** Reversible pS129 triggered by neuronal activity in DIV17-21 rat cortical neurons as indicated in the schematic (20 µM PTX, 1 µM TTX). WB for total αS and pS129. *N* = 4 independent experiments on different days, *n* = 16 biological replicates total; ****p < 0.0001. Mean +/− SD. Brown-Forsythe and Welch ANOVA with Dunnett’s T3 post hoc test for multiple comparisons. **f** Kinetics of reversible pS129 in DIV17-21 rat cortical neurons as indicated in the schematic (20 µM PTX, 1 µM TTX). WB for total αS and pS129. *N* = 5 independent experiments on different days, *n* = 15 biological replicates total; *p < 0.1; ***p < 0.001. Mean +/− SD. Brown-Forsythe and Welch ANOVA with Dunnett’s T3 post hoc test for multiple comparisons.

Having established activity-dependent pS129 in vivo by a physiologically relevant stimulus, we then sought to address the pathways that lead to physiological pS129 increase in cultured neurons. First, we assessed the specificity of the phosphorylation site in αS. Upon 2 h of PTX stimulation of primary rat cortical neurons, we observed a pronounced increase in pS129, whereas levels of pY39, pY125, and pY136 were unaltered (Fig. 2a–d), consistent with a specific, regulated event. We reasoned that fast reversibility of activity-dependent pS129 would be another indication of physiological relevance. In an initial “wash-out” experiment, rat cortical neurons were exposed to 20 µM PTX for 2 h followed by a switch to fresh PTX-free medium for 15, 30, 60, or 120 min. A significant reduction (~20%) in pS129 levels occurred as early as 15 min after medium replacement, but basal levels were not reached after 2 h, indicating that neuronal stimulation may persist upon wash-out (data not shown). We therefore tested for reversibility by fully blocking ongoing neuronal activity and compared 2 h PTX with 4 h PTX or 4 h PTX with TTX added halfway through PTX treatment (i.e., 2 h after PTX addition). The pS129 levels in PTX/TTX-treated neurons were only slightly higher than in DMSO-treated controls and markedly lower than in of neurons treated with 2 or 4 h of PTX (Fig. 2e). To probe further the kinetics of pS129 reversal after stimulation, we treated stimulated neurons (2 h PTX) with TTX for 15, 30, 60, or 120 min (Fig. 2f). pS129 levels were significantly reduced 15 min after blocking stimulation, and by 120 min, pS129 levels were indistinguishable from basal levels. Together, these findings were consistent with specific, dynamic, and reversible activity-dependent αS S129 phosphorylation.

### Activity-dependent pS129 is mediated by Plk2, PP2A, calcineurin, and calcium influx

**Fig. 3:**
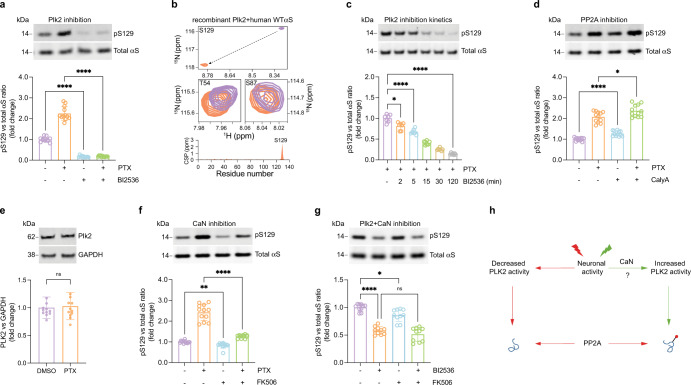
Activity-dependent pS129 is regulated by Plk2, PP2A, and calcineurin. **a** DIV17-21 rat cortical neurons treated with 20 µM PTX, 500 nM BI2536 (Plk2 inhibitor) or PTX + BI2536 for 2 h. WB for total αS and pS129. *N* = 3 independent experiments on different days, *n* = 12 biological replicates total. *p < 0.1; ****p < 0.0001. Mean +/− SD. Brown-Forsythe and Welch ANOVA with Dunnett’s T3 post hoc test for multiple comparisons. **b** 2D ^1^H-^15^N HSQC NMR spectrum of recombinant, N-terminally acetylated human αS in the presence (orange) or absence (purple) of recombinant Plk2 (see “Methods” for details and Supplementary Fig. 4). Spectrum in the presence (purple) or absence of Plk2 (orange) is shown for S129 (top), T54, and S87 (middle); residue-resolved combined chemical shift perturbations (CSPs) of backbone amide resonances (bottom). **c** DIV17-21 rat cortical neurons treated with 20 µM PTX for 2 h and 500 nM BI2536 co-administered for 0, 2, 5, 15, 30, or 120 min. WB for total αS and pS129. *N* = 2 independent experiments on different days, *n* = 7 biological replicates total. *p < 0.1; ****p < 0.0001. Mean +/− SD. Brown-Forsythe and Welch ANOVA with Dunnett’s T3 post hoc test for multiple comparisons. **d** 1.5 nM PP2A inhibitor calyculin A (Caly A) treatment for 2 h. WB for total αS and pS129. *N* = 3 independent experiments on different days, *n* = 12 biological replicates total. ***p < 0.1; ****p < 0.0001. Mean +/− SD. Brown-Forsythe and Welch ANOVA with Dunnett’s T3 post hoc test for multiple comparisons. **e** DIV17-21 rat cortical neurons treated with 20 µM PTX or DMSO control for 2 h. WB for PLK2 and GAPDH (loading control). *N* = 3 independent experiments on different days, *n* = 12 biological replicates. ns not significant. Mean +/− SD. Unpaired *t*-test, Welch’s correction; two-tailed. **f** DIV17-21 rat cortical neurons treated with DMSO, 20 µM PTX, 2 µM CaN inhibitor FK506, or both for 2 h. WBs to total αS and pS129. *N* = 3 independent experiments on different days, *n* = 12 biological replicates total. **p < 0.01; ****p < 0.0001. Mean +/− SD. Brown-Forsythe and Welch ANOVA with Dunnett’s T3 post hoc test for multiple comparisons. **g** DIV18 rat cortical neurons treated with 50 nM Plk2 inhibitor BI2536, 2 µM CaN inhibitor FK506, or both for 2 h. WB for total αS and pS129. *N* = 3 independent experiments on different days, *n* = 10 biological replicates total. ns not significant; **p < 0.01; ****p < 0.0001. Mean +/− SD. Brown-Forsythe and Welch ANOVA with Dunnett’s T3 post hoc test for multiple comparisons. **h** Schematic: the molecular cascade leading to activity-dependent pS129 (see main text for details).

While mainly implicated in αS phosphorylation under pathological conditions to date^27^, based on a previous study Plk2 was a candidate kinase for catalyzing the observed activity-dependent αS phosphorylation at Serine129^28^. Accordingly, we quantified levels of total αS and pS129 in rat cortical neurons treated with either vehicle alone (DMSO), PTX, BI2536 (a potent Plk2 inhibitor)^11^, or PTX plus BI2536 (Fig. 3a). As before, PTX markedly increased pS129 over basal levels. Strikingly, BI2536 virtually abolished not only the activity-dependent but also the basal pS129 levels, indicating Plk2 is the major αS Serine129 kinase in cortical neurons. To test the substrate-enzyme relationship between Plk2 and pS129 in a reductionist system, we compared the NMR spectra of recombinant human αS to those of recombinant αS incubated with Plk2 and all relevant cofactors for the enzymatic reaction (see Methods; Fig. 3b and Supplementary Fig. 4). The only substantial difference between the two spectra was a strong shift for amino acid S129 upon incubation with Plk2, confirming that highly specific S129 phosphorylation had occurred (Fig. 3b top and bottom panels). Other potential serine/threonine phosphorylation sites such as S87 (Fig. 3b middle right panel) or T54 (Fig. 3b middle left panel) were not affected.

We next assessed the kinetics of S129 phosphorylation by Plk2 in neurons. We observed a significant reduction in pS129 as early as 2 min after Plk2 inhibition, and a 50% reduction after ~15 min (Fig. 3c), suggesting that a relatively fast-acting phosphatase balances the effect of Plk2. A candidate for that role was protein phosphatase 2A (PP2A) based on a prior study^29^. Adding the PP2A inhibitor calyculin-A (CalyA)^30^ to unstimulated neurons caused a small but significant increase in pS129 levels after 2 h (Fig. 3d). CalyA addition to PTX-stimulated neurons further increased pS129 relative to PTX alone, suggesting that PP2A counteracts Plk2-dependent S129 phosphorylation under both basal and stimulated conditions (Fig. 3d). Based on these findings, we assumed an increase in Plk2 protein levels might be a possible mechanism of activity-dependent pS129, consistent with earlier studies reporting increased Plk2 levels upon 24 h of PTX stimulation of neurons^23,31^. However, the pronounced pS129 elevation that we observed after only 2 h of stimulation was not accompanied by elevated Plk2 protein levels (Fig. 3e), suggesting that Plk2-dependent pS129 might be regulated via a posttranslational mechanism. A synaptic protein that is well-known to be regulated by neuronal activity via Ca^2+^/calmodulin is the phosphatase CaN. Strikingly, when we added the CaN inhibitor FK506 to the neurons, pS129 levels were reduced both in the presence and absence of PTX stimulation (Fig. 3f). We found that the pS129-reducing effect of Plk2 inhibition was not augmented further by CaN inhibition (i.e., there were no additive effects) (Fig. 3g), suggesting that CaN and Plk2 may act in the same pathway, with CaN likely to be upstream of Plk2.

Overall, our findings were consistent with the following model (Fig. 3h). (i) Under basal conditions including spontaneous network activity, Plk2 is moderately active, thereby continuously generating pS129 that undergoes constant removal of the phosphate group by PP2A. (ii) Neuronal stimulation elevates cellular Ca^2+^ levels, which activate CaN. CaN increases Plk2 activity by a posttranslational mechanism, possibly by dephosphorylation of Plk2. This increased enzyme activity elevates pS129 because it outpaces basal PP2A activity. (iii) If neuronal activity, CaN, or Plk2 are blocked, PP2A activity prevails, and pS129 levels are minimal. Consistent with the notion that elevated Ca^2+^ levels and CaN activation are upstream of Plk2, we found antagonists to L-, N-, or P/Q- type calcium channels to prevent activity-dependent pS129 to variable degrees, with the strongest effect seen with the L-type channel blocker nimodipine (Supplementary Fig. 5c–e).

### Activity-dependent pS129 localizes to membranes and synaptic boutons

**Fig. 4:**
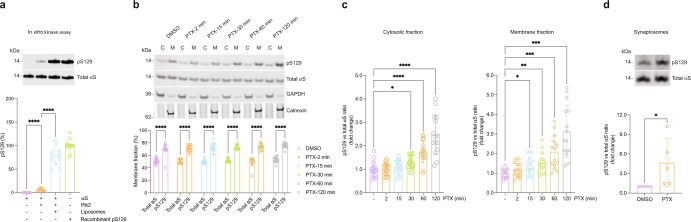
Activity-induced pS129 is enriched in membrane fractions. **a** In vitro kinase assay was carried out by incubating 2 µM recombinant αS alone, αS + 2 µM Plk2, and αS + Plk2 + 0.2 mM liposomes. *N* = 3 independent experiments on different days, *n* = 12 biological replicates total; ****p < 0.0001. Mean +/− SD. RM one-way ANOVA with post hoc Sidak’s multiple comparisons test. **b** DIV18-20 rat cortical neurons treated with 20 µM PTX for the indicated time points and subjected to sequential extraction to generate cytosol (C) vs. membrane (M) protein lysates. WB for total αS and pS129. Quantification of total αS and pS129 in membrane fractions. *N* = 4 independent experiments on different days, *n* = 16 biological replicates total. ****p < 0.0001. Mean +/− SD. Two-way ANOVA with post hoc Tukey’s multiple comparisons test. **c** Samples as in **b**. Quantification of total αS vs pS129 ratios as indicated in cytosolic and membrane fractions, respectively. *p < 0.05; **p < 0.01; ***p < 0.001; ****p < 0.0001. Mean +/− SD. Brown-Forsythe and Welch ANOVA with Dunnett’s T3 post hoc test for multiple comparisons. **d** DIV18-21 rat cortical neurons treated with DMSO or 20 µM PTX followed by isolation of synaptosomes. WBs for total αS and pS129. *N* = 6 independent experiments on different days. *p < 0.04. Mean +/− SD. Ratio paired *t*-test; two-tailed.

αS is known to bind to liposomes by forming amphipathic helices, similar to its binding to synaptic vesicles^32,33^. To further understand the effect of Plk2 in regulating pS129, we employed a reductionist system and incubated recombinant human αS and Plk2 proteins (plus cofactors; see “Methods”) in the presence or absence of liposomes. We observed S129 phosphorylation upon incubation with Plk2, but this was markedly greater in the presence of liposomes, indicating that helical αS at membranes is an optimal Plk2 substrate (Fig. 4a).

To study the cytosol vs. membrane distribution of activity-induced pS129 relative to total αS (and potential changes of that distribution over time), we treated rat cortical neurons with 20 µM PTX for 0, 2, 15, 30, 60, and 120 min. We then subjected the cells to centrifugation-free sequential digitonin extraction^34^ to separate cytosolic from membrane proteins (Fig. 4b). Immunoblotting for calnexin, a transmembrane protein, and the soluble enzyme GAPDH confirmed the relative purity of the fractions prepared by this method (Fig. 4b). Consistent with the ability of αS to transiently interact with membranes as shown in our previous work^34^, total αS was recovered to a similar extent in cytosolic (C) and membrane (M) fractions, and this ~50:50 C:M distribution did not change significantly in the course of neuronal stimulation (Fig. 4b). pS129, in contrast, was enriched in the membrane fraction (~30:70 C:M distribution), both under basal conditions and during the time course of PTX stimulation (Fig. 4b). Interestingly, we found that activity-dependent pS129 may occur faster in membrane-associated αS. In the membrane fraction, a significant increase in pS129 was observed as early as 15 min after PTX stimulation (Fig. 4c, left panel) but in the cytosolic fraction only after 30 min (Fig. 4c, right panel). The ~30:70 C:M distribution remained largely unchanged over time, as pS129 levels in both cytosol and membrane fractions rose in parallel (Fig. 4b). Similarly, the C:M ratio of total αS did not change over time (Fig. 4b), ruling out that a gross redistribution of αS from cytosol to membrane fractions might trigger the activity-dependent rise in pS129. We then asked whether pS129 status regulates the membrane interaction of αS by fractionating *SNCA*^*−/−*^ rat neurons expressing exogenous rat αS WT or αS S129A (phospho-incompetent mutant) both under basal and stimulated conditions. Strikingly, the membrane interaction was not affected by pS129 status since the levels of membrane-associated αS S129A were indistinguishable from those of αS WT either with or without stimulation by PTX (Supplementary Fig. 7).

**Fig. 5:**
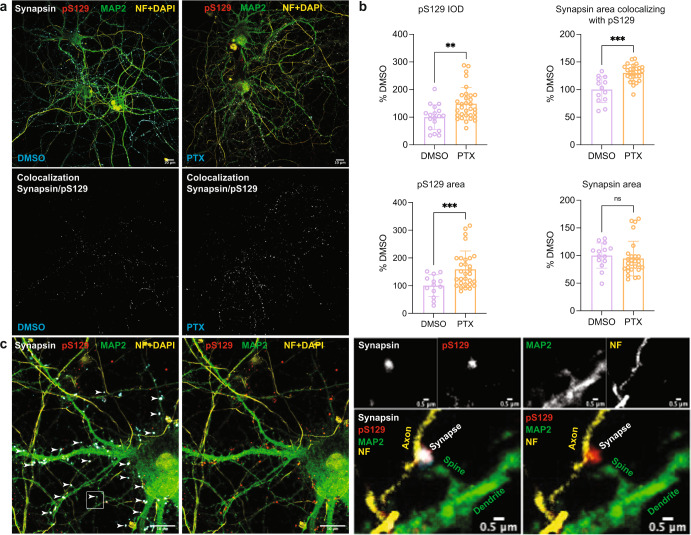
Increased pS129 signal and localization in synapsin-containing boutons after PTX stimulation. **a** Representative images of synapsin/pS129 colocalization in DIV18 rat cortical neurons treated with DMSO vs. 20 µM PTX. Top images: synapsin, pS129, MAP2, and neurofilament (NF) + DAPI staining. DMSO control on the left, PTX treatment on the right. Bottom images: colocalization mask synapsin/pS129. DMSO control on the left, PTX treatment on the right. Scale bar, 10 μm. **b** Quantifications (DMSO vs. PTX). Top left, pS129 integrated optical density (IOD). Quantification of *N* = 14 images for DMSO; *N* = 30 images for PTX. Top right, synapsin area colocalizing with pS129. *N* = 13 images for DMSO; *N* = 29 images for PTX. Bottom left, pS129 area. *N* = 13 images for DMSO; *N* = 30 images for PTX. Bottom right, synapsin area. *N* = 14 images for DMSO; *N* = 30 images for PTX. Each image contained ~3–5 cells. **p < 0.01; ***p < 0.001; ns not significant. Mean ± SD. Unpaired *t*-tests with Welch’s correction; two-tailed. **c** Localization of pS129 at synapsin-immunopositive synapses in PTX-stimulated neurons. Arrowheads indicate synapses labeled for both pS129 and synapsin. All visualized images represent maximized 3D projection of 0.9 µm thick z-stacks. Scale bars in the left images, 10 µm; scale bars in the right images, 0.5 µm. Magnifications on the right: specific colocalization of pS129 with synapsin at presynaptic terminals in contrast to MAP2 positive dendritic profiles.

In mature neurons, a large fraction of αS associates with or is in proximity of synaptic vesicles^16^. Therefore, we determined the pS129 status of the synaptic vesicle-associated αS fraction by isolating synaptosomes from PTX- and control-treated rat cortical neurons. Indeed, we observed abundant αS levels and PTX-dependent pS129 accumulation, suggesting that pS129 accumulates at least in part at the membranes of synaptic vesicles (Fig. 4d). Next, we sought to study the subcellular localization of basal and activity-induced pS129 in more detail. High-resolution immunofluorescent confocal microscopy of PTX-treated and untreated neurons revealed a marked overlap between pS129-positive foci and the presynaptic protein synapsin under both basal and stimulated conditions while their overlap with adjacent MAP2-positive dendritic profiles was limited (Fig. 5a–c). As expected, pS129 signals were increased upon PTX treatment (synapsin area was unchanged; Fig. 5a, b). Synapsin/pS129 co-localization was already apparent in the absence of PTX and was enhanced by stimulation (Fig. 5a, b). This result was consistent with the strong αS total and pS129 immunoreactivity that we had observed biochemically in synaptosomes (Fig. 4d). A lack of pS129-specific signal in rat *SNCA*^−/−^ neurons but specific pS129 synaptic signals in rat *SNCA*^+/+^ (WT) neurons using antibodies EP1536Y and D1R1R further validated our observation (Supplementary Fig. 6). To our knowledge, this is the first time pS129 localization at synapses has been documented under explicitly physiological conditions.

### Serine129 phosphorylation positively regulates neurotransmission

**Fig. 6:**
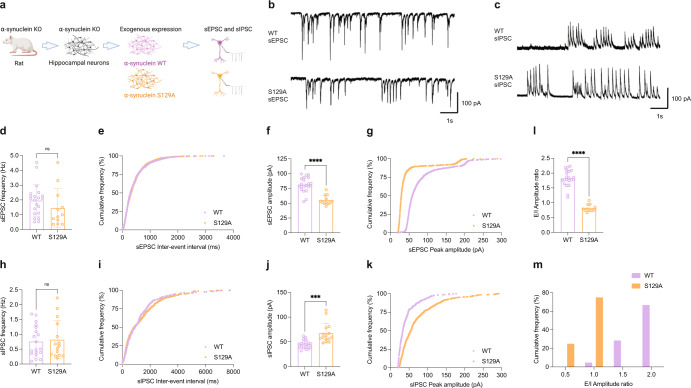
Phospho-deficient S129A αS reduces the excitatory/inhibitory balance in rat *SNCA*^*−/−*^ hippocampal neurons. **a** Spontaneous excitatory (sEPSC) and inhibitory postsynaptic currents (sIPSC) measured in DIV14-18 hippocampal neurons from *SNCA*^*−/−*^ rats transduced with αS WT or αS S129A (schematics created with BioRender.com). **b** Representative sEPSC traces. **c** Representative sIPSC traces. Scale bars, *X*-axis = 1 s and *Y*-axis = 100 pA. **d** sEPSC frequency in *SNCA*^*−/−*^ neurons expressing WT αS or αS S129A. **e** Cumulative frequency distribution of data shown in **d**, expressed as percentage. **f** sEPSC amplitude in *SNCA*^*−/−*^ neurons expressing αS WT or αS S129A. **g** Cumulative frequency distribution plot of data shown in **f**, expressed as percentage. Total number of individual events analyzed in panels **e** and **g**: αS WT = 1228; αS S129A = 1701. Total number of individual cells across two independent neuronal cultures (*N = 2*) recorded in sEPSC experiments: WT αS = 21 and αS S129A  *n* = 12. **h**, **i** sIPSC frequency between conditions as bar charts and cumulative distribution, respectively. **j** sIPSC amplitude in *SNCA*^*−/−*^ neurons expressing WT or S129A αS. **k** Cumulative frequency distribution of data shown in **j**. Total number of individual events analyzed in panels **i** and **k**: αS WT = 302; αS S129A = 585. Total number of individual cells across two independent neuronal cultures recorded in sIPSC experiments: αS WT = 19 and αS S129A = 15. **l**, **m** The excitatory/inhibitory ratio of the amplitude as a bar chart and cumulative frequency distribution, respectively. E/I amplitude ratios were derived from **f** and **j**. Respective averages in **f** (αS WT or αS S129A) were divided by respective individual values (αS WT or αS S129A) in **j** to obtain E/I amplitude ratios. Each circle represents an individual cell. Recordings performed on at least three different days. Unpaired *t*-tests with Welch’s correction; two-tailed; mean ± SD; ns not significant; ***p < 0.001; ****p < 0.0001.

The role of αS at the synapse remains unclear. Several functions have been ascribed to αS, ranging from limiting synaptic vesicle movement^35^, regulating the kinetics of synaptic vesicle endocytosis^36^, to controlling the opening of the fusion pores that vesicles form at the synaptic cleft^37^. Since we found neuronal activity to induce pS129, we sought to test in hippocampal neurons whether phosphorylation of Serine 129 has an effect on neuronal excitability. Hippocampal neurons are more homogenous^38^ and proved to be superior to cortical neurons in our electrophysiological experiments with regard to their robustness. Given that our collective biochemical evidence had been obtained in cortical neurons, we first confirmed that activity-dependent pS129 accumulation also occurs in hippocampal neurons (Supplementary Fig. 8a). We then employed whole-cell patch clamping to measure both spontaneous excitatory and inhibitory postsynaptic currents (sEPSC and sIPSC) specifically in pyramidal (glutamatergic) neurons. To this end, we introduced exogenous expression of rat αS in *SNCA*^*−/−*^ rat hippocampal neurons via lentiviral transduction. In addition to the WT protein, we also assessed the expression of S129A that cannot be phosphorylated at position 129 (Fig. 6a). The bicistronic system enabled the expression of untagged αS variants along with ZsGreen1, a fluorescent reporter. Pyramidal neurons expressing ZsGreen1 were identified, patched, and recorded. αS WT and αS S129A were expressed at closely similar levels (Supplementary Fig. 8b). We found the frequencies of both sEPSC (Fig. 6d, e) and sIPSC (Fig. 6h, i) to be unaltered between neurons expressing these two variants. In contrast, the respective amplitudes, which are mediated by postsynaptic receptor activation/inhibition, were significantly different between αS WT and αS S129A. In the case of sEPSCs, we observed a significant reduction in *SNCA*^−/−^ neurons exogenously expressing αS S129A relative to *SNCA*^*−/−*^ neurons complemented with αS WT (Fig. 6f, g). In the case of sIPSCs, conversely, we observed an increase in αS S129A-expressing relative to αS WT-expressing neurons (Fig. 6j, k). Consequently, the sEPSC/sIPSC (E/I) amplitude ratio was markedly reduced by S129A (Fig. 6l, m). Representative traces for these single-cell recordings illustrate the findings further (Fig. 6b, c). Together, our observations suggested a relative inhibitory role of phospho-deficient αS and, therefore, an excitatory role of pS129 in neurotransmission.

### Synaptic plasticity is impaired in S129A^KI^ αS mice

**Fig. 7:**
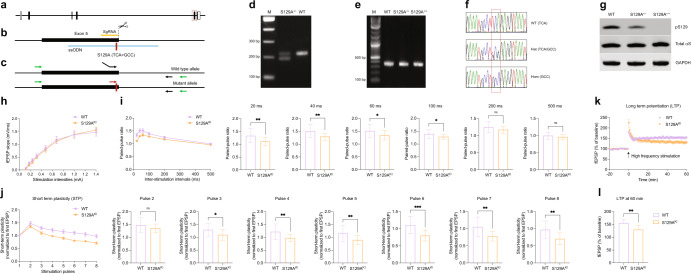
Synaptic plasticity is impaired in S129A^KI^ mouse. **a**–**f** Generation of the S129A knock-in mutation in mice. **a** Genomic structure of the mouse *SNCA* gene. Exons are depicted after transcript variant *SNCA-201* (ENSMUST00000114268.5), with coding and non-coding regions shown as filled or open boxes, respectively. Exon 5 is boxed with red dashed line. **b** A knock-in strategy using CRISPR-Cas9 and ssODN was employed to generate the S129A mutation in Exon 5 of the endogenous *SNCA* gene. Relative positions of sgRNA (orange horizontal line), ssODN (blue horizontal line), and the S129A mutation (red vertical line) are indicated. **c**–**f** ES cell clone screening and mouse genotyping. A 3-primer PCR strategy (primers indicated with black and red arrows) was designed to distinguish WT and mutant alleles (**c**, **d**). For genotyping, a common pair of primers (indicated with green arrows) was used for PCR followed by sequencing to distinguish different genotypes (**c**, **e**, **f**). **g** Total mouse brain homogenates from indicated genotypes. WB for total αS, pS129 (D1R1R) and GAPDH. **h** Input and out current curve from hippocampal slices (CA1 region) of indicated mouse genotypes. *N* = 3 animals each, *n* = 16 (WT) or 17 (S129A^KI^) individual slices. **i** Paired-pulse facilitation of WT and S129A^KI^ hippocampal slices. Inter-stimulation intervals as indicated. *N* = 3 animals each, *n* = 16 (WT) or 17 (S129A^KI^) individual slices. Unpaired *t*-tests for 20, 40, 60, 100, 200, and 500 ms with Welch’s correction; two-tailed; mean ± SD; ns not significant; *p < 0.05; **p < 0.01. **j** Short-term plasticity assessed by multi-pulse events. Values normalized to first excitatory post synaptic current (EPSP). *N* = 3 animals each, *n* = 16 (WT) or 17 (S129A^KI^) individual slices. Unpaired *t*-tests for pulses 2, 3, 4, 5, 6, 7, and 8 with Welch’s correction; two-tailed; mean ± SD; ns not significant; *p < 0.05; **p < 0.01; ***p < 0.001. **k**, **l** Long Term Potentiation (LTP) of WT and S129A^KI^. LTP induced by standard 100 Hz stimulation. *N* = 3 animals each, *n* = 8 (WT) or 10 (S129A^KI^) individual slices. Unpaired *t*-tests for panel **l** (values at 60 min from K) with Welch’s correction; two-tailed; mean ± SD; **p < 0.01.

We extended our in vitro findings to in vivo by first generating an S129A^KI^ αS mouse model via CRISPR-mediated homology-directed repair (Fig. 7a–f). pS129 signals were completely abolished in S129A^+/+^ (S129A^KI^) homozygous mice while in S129A^+/−^ heterozygous mice pS129 was greatly reduced compared to that of WT animals as revealed by WB (Fig. 7g). Age-matched male mice were then used throughout for field recording experiments in the CA1 regions of hippocampal slices. We first measured the input/out curves in WT and S129A^KI^ αS mice. Stimulation intensities within a range of 0.1 to –1.4 mA revealed no obvious differences across currents in S129A^KI^ αS vs. WT mice (Fig. 7h). Next, to explore whether activity-dependent pS129 regulates the presynaptic release function, we studied paired-pulse facilitation (PPF) by applying two stimuli in quick succession with an inter-stimulation interval ranging from 20 to 500 ms. We found PPF to be significantly impaired in S129A^KI^ αS mice compared to WT mice at intervals between 20 and 100 ms. Beyond these inter-stimulation intervals, PPF was largely indistinguishable between WT and S129A^KI^ αS mice (Fig. 7i). We next asked whether short-term plasticity (STP) was altered in S129A^KI^ αS mice. Hippocampal slices were stimulated 8 times at 40 ms intervals with each stimulus containing pulses at 25 Hz. While the first two recorded EPSPs were not different between WT and S129A^KI^ αS mice, amplitudes of consecutive EPSPs were significantly decreased in S129A^KI^ αS mice (Fig. 7j). Finally, we recorded LTP in these mice. To induce LTP, two consecutive trains of 1 s stimuli were given at 100 Hz separated by 20 s in the CA1 region of hippocampal slices (for additional details, see Methods). Similar to STP, we also observed a significant impairment in LTP in S129A^KI^ αS mice (Fig. 7k, l). Thus, in situ recordings from S129A^KI^ αS mice together with in vitro patch clamp analyses of cultured neurons supported the notion that activity-dependent phosphorylation of αS at S129 enhances synaptic transmission.

## Discussion

Our work reveals a pronounced, site-specific, and reversible phosphorylation of αS Serine129 that occurs physiologically during neuronal activity. We employed conditions that we considered as close to physiological as possible (endogenous wt αS; neuronal stimulation under conditions described by widely used protocols in the absence of cytotoxicity; environmental enrichment in non-transgenic mice) to study normal, non-pathological events. A detailed characterization of phosphorylation related to its normal function, particularly at synapses, has not previously been undertaken. Our study unearths a physiological role for pS129 in fine-tuning excitatory/inhibitory balance during neuronal activity, to wit: (1) pS129 occurs normally in response to synaptic activity in vitro (cortical and hippocampal neurons) and in vivo (animals under EE vs. SH); (2) activity-dependent pS129 is reversible and not associated with toxicity; (3) other αS phosphorylation sites are not altered, (4) activity-dependent pS129 is dependent on Plk2, with the involvement of PP2A and CaN; (5) activity-dependent pS129 is enriched at membranes and specifically in presynaptic boutons; (6) activity-dependent pS129 serves to fine-tune the balance between excitatory and inhibitory neuronal activity; and (7) activity-dependent pS129 positively regulates synaptic plasticity in mice.

We observed physiological, activity-dependent pS129 induction in cultured neurons by lowering inhibitory tone using well-validated neuropharmacological methods (PTX; BIC)^18,23–25^. In living animals, we employed EE, a ‘natural’ stimulus known to be mediated in part by enhanced glutamatergic neurotransmission^26,39^. Thus, different forms of neuronal activation elevated pS129 levels. Our finding that Plk2 inhibition virtually reduces pS129 to zero (Fig. 3a) suggests that both basal and activity-dependent pS129 pools are Plk2-dependent. We further showed that interrupting synaptic activity with TTX lowers pS129 levels by ~25%, but does not fully block it (Fig. 1b). Thus, even in the absence of any neuronal stimulation, basal Plk2 activity may be sufficient to counteract the continuous de-phosphorylation of S129 by PP2A (Fig. 3d).

αS undergoes multiple post-translational modifications either individually or in combination under both normal and pathological conditions (reviewed in ref. ^40^). We found that phosphorylation of Y39, Y125, and Y136 were not influenced by neuronal activity (Fig. 2b-d). Human αS has been suggestted to be constitutively phosphorylated at residue S129 and, to a lesser extent, S87^41^. Because both the in vitro and in vivo approaches in our study relied on rodent models, S87, another important phospho-site could not be addressed: in rodents (both rats and mice), asparagine, not serine, is found at position 87. In future work, it will be interesting to study (i) S87 phosphorylation in response to neuronal activity in human neurons; (ii) any additional activity-dependent changes in αS post-translational modifications by unbiased methods such as mass-spectrometry; and (iii) any activity-dependent structural changes in rodent and human αS by unbiased approaches such as in-cell NMR^42,43^.

There is no consensus on what governs pS129 under pathological conditions: besides Plk2, different kinases have been proposed to be involved, and pS129 has been described to be upstream of, downstream of, or unrelated to αS aggregation (reviewed in ref. ^40^; also addressed in a recent study^44^). A parsimonious interpretation of how physiological pS129 may be regulated in neurons is summarized in Fig. 3h: activity-triggered Ca^2+^ elevation activates CaN, which in turn increases Plk2 activity via a post-translational mechanism. Plk2 then phosphorylates αS at Serine129. This reaction is counteracted by continuous S129 dephosphorylation by PP2A. We further established that Plk2 may have a preference for vesicle-associated, helically folded αS (Fig. 4a). This is consistent with our observation that the kinetics of activity-dependent pS129 may be faster in membrane than in cytosolic fractions (Fig. 4c, d). Interestingly, it has been proposed that Ca^2+^ binding to the αS C-terminus increases αS-membrane interactions^45^. However, we did not observe an activity-dependent redistribution of αS from the cytosol to vesicle membranes as a trigger for pS129 (Fig. 4b). Our data suggest that the interaction between αS and membranes enhances pS129 by Plk2, which leads to the enrichment of pS129 in membrane fractions; however, pS129 status itself does not affect the interaction of αS with membranes (Fig. 4a and Supplementary Fig. 7). Thus, even though activity-dependent changes in Ca^2+^ are very likely to be involved in the regulation of pS129, our data do not support a mechanism in which Ca^2+^ elevation increases αS-membrane binding as a trigger for rising levels of pS129. However, CaN, postulated to be upstream of Plk2 in the pathway that we propose, is activated by Ca^2+^ and calmodulin binding^46,47^. Based on these observations, it would not be unexpected to find that any elevation of cytosolic Ca^2+^ levels may increase pS129 (in neurons, activity and cytosolic Ca^2+^ elevation is difficult to separate). Whether and how pS129 is altered in response to a variety of triggers (and the consequences on neuronal function) will be of interest in follow-up studies. Of note, Plk2 activation has been proposed to play a critical role in homeostatic synaptic plasticity during elevated activity^23,31^, so whether pS129 has a function in synaptic scaling remains to be explored.

In our experiments addressing the functional relevance of pS129 by whole-cell patch clamp recording, we observed a reduction of sEPSC amplitudes in neurons expressing phospho-deficient S129A relative to WT αS (Fig. 6f, g). sIPSCs changed in the opposite direction (Fig. 6j, k). As a result, S129A markedly reduced the sEPSC/sIPSC (E/I) amplitude ratio (Fig. 6l, m). These observations were consistent with a relative inhibitory role of unphosphorylated S129 αS and, therefore, an excitatory role of pS129. Our data suggest that phosphorylating Serine129 reduces the inhibitory effects of αS WT on synaptic activity. Thus, increases in pS129 during excitatory firing seem to amplify neuronal activity further, consistent with a feed-forward mechanism. pS129 has been proposed to be a trigger for selective autophagosomal αS degradation under pathological conditions^11^, which could explain decreased αS function upon pS129 increase. However, under the conditions that we tested, no evidence for αS level reduction was observed when pS129 was induced by neuronal activity. While pS129 was increased more than 100% upon 2 h and 350% after 6 h of PTX treatment of cultured neurons (Fig. 1a and Supplementary Fig. 2a, b), αS levels did not decrease then or even after 24 h of PTX (Fig. 1d and Supplementary Fig. 3a, c). Thus, our data suggest this post-translational modification does not alter αS stability. However, it remains to be seen whether accumulation or a lack of pS129 for a prolonged duration will affect endogenous αS protein turnover. In our experimental setup, pS129 may affect αS function by either altered localization or altered interaction with binding partners (lipids, other proteins).

Several synaptic plasticity parameters were found to be affected in hippocampal slices from S129A^KI^ vs. WT mice (Fig. 7). Because PPF and STP seem to be impaired in S129A^KI^ mice, pS129 may regulate the presynaptic release of glutamate related to hippocampal plasticity. The observed LTP impairment may be explained by STP impinging on LTP. Given that the elevation of Ca^2+^ is crucial for PPF^48,49^ and calcium channel blockers differentially affected activity-dependent pS129 (Supplementary Fig. 5c–e), the LTP impairment in S129A^KI^ mice may not be surprising. We speculate that pS129 might fine-tune the recycling of synaptic vesicle (endocytosis) during neurotransmission^36,50,51^. Alternatively, pS129 could play a role in one or many of the steps in exocytosis such as (a) synaptic vesicle clustering^52,53^, (b) mobility of synaptic vesicles^35^, (c) synaptic vesicle fusion and SNARE complex assembly^54,55^, and (d) dilation of the fusion pore^37,55^. In addition, the possibility of a post-synaptic mechanism should not be ignored. The presence of pS129 is obvious in presynaptic boutons, probably due to a tight association of this species with synaptic vesicles, but its diffused distribution along dendrites suggests pS129 can also be found in post-synaptic components. Given that Plk2 is a master regulator of homeostatic plasticity^23,31,56,57^ and involved in AMPA receptor trafficking^58,59^, it is tempting to speculate that activity-dependent pS129 might play a role in synaptic scaling. Interestingly, the modulators of activity-dependent pS129 such as Plk2, CaN, and L-type calcium channels all are typically considered to be enriched at the post-synaptic side^23^. Future work will shed light on the exact role(s) of pS129 at neuronal synapses.

Early characterizations of LBs revealed that ~90% of αS in these lesions is phosphorylated at S129^1^. Thus, our new observations are consistent with a dual role of pS129 in health and disease: a normal role in regulating the synaptic homeostasis, and a pathological role in the aggregation and deposition of insoluble αS into Lewy bodies and neurites. Our data suggest that membrane-associated αS is a better substrate for Plk2 than soluble αS (Fig. 4a). In an analogous fashion, aggregated αS might be a better substrate than soluble, dynamic αS. Our discovery of a dynamic phosphorylation-dephosphorylation and a normal physiological function of pS129 raises the question of whether this dynamic nature becomes deregulated under pathological conditions. Reports that even in diseased brain, pS129 can be found in soluble fractions^60^ may indicate that excess prefibrillar αS is phosphorylated under pathological conditions. However, in a recent study, phosphorylation of serine-129 was shown to occur after the initial protein deposition^61^. In light of the latter report, it might be possible to distinguish between pathological pS129 (late event on deposited αS) and physiological pS129 (part of normal αS homeostasis in soluble and membrane-associated synaptic fractions, no signs of aggregation). In other words, dynamic synaptic pS129 may not be upstream of pathological pS129 in aggregates, but unrelated. Alternatively, elevated pS129 in PD and DLB may represent an attempt by neurons to enhance neuronal excitability and keep electrophysiological function intact in the face of the steady dysfunction and loss of synapses and neuronal death during synucleinopathies. While our study suggests that increased pS129 by neuronal activity in hippocampal neurons is a physiological response, our finding could be of potential relevance in the context of synucleinopathy. Hippocampal αS pathology has been implicated in the occurrence of non-motor symptoms in PD, because (a) the clinical diagnosis of dementia in PD patients is associated with an increased burden of hippocampal Lewy pathology^62^; (b) increased levels of insoluble (pS129) αS in the hippocampus^63,64^; (c) compromised hippocampal plasticity plays a vital role in non-motor symptoms of PD^65,66^; (d) impaired LTP is reported in multiple pre-clinical models of PD^67,68^. For further appraisal of the potential relevance of activity-dependent pS129 to PD, it will be important to investigate the role of activity-dependent pS129 in dopaminergic signaling.

Moreover, we speculate that physiological pS129 may have been underestimated because some or much of it may be dephosphorylated by PP2A and other phosphatases in the agonal period or after death. In contrast, pS129 which is associated with pathological structures may be protected in synucleinopathy brain from post-mortem dephosphorylation as large proteinaceous aggregates render them inaccessible to phosphatases and other degrading enzymes^69^, and/or their association with lipids and organelle structures^70,71^. Of note, our work on physiological pS129 is thematically connected to emerging concepts in the tau field that the role of phosphorylated tau is not only associated with pathology but also normal physiology^72^. Taken together our findings have major implications not only for understanding the normal function of αS at the neuronal synapses but also reinterpreting the widely cited accumulation of pS129 as a key event in PD and dementia with Lewy bodies.

## Methods

### Animal approval

All animal procedures were approved by the Institutional Animal Care and Use Committee at BWH.

### Generation of S129A^KI^ αS mouse

The S129A mutation in the mouse *SNCA* gene (NCBI Gene ID: 20617) was generated using CRISPR-mediated homology-directed repair (HDR) in a mouse embryonic stem (ES) cell line of the 129S6 × C57BL/6N origin (Leveragen). A single-stranded oligodeoxynucleotide (ssODN) template was designed to contain a point mutation (underlined) which substitutes Serine (TCA) at the amino acid position 129 in Exon 5 of *SNCA* with Alanine (GCC), and additional 50 nucleotides flanking the S129A mutation (5′-TCCTGGAAGACATGCCTGTGATCCTCATGAGGCTTATGAAATGCCTGCCGAGGTAAATGCCTGTATAAAGAAAACTAAGCAAAACACTTTAGGTGTTTAATTT-3′). A sgRNA oligo targeting Exon 5 (5′ GGCTTATGAAATGCCTTCAG-3′) was cloned into a U6-sgRNA expression vector, which was co-transfected with a Cas9 expression vector and a Puromycin expression vector into ES cells using Lipofectamine LTX (ThermoFisher) according to the instructions of the manufacturer. Puromycin selection (5 mg/ml) was applied 24 h after transfection for 2 days to enrich for transfectants. Correctly targeted ES cell clones were identified by a 3-primer PCR with 5′-gcagtaccactaaggcagagtaagtCAGTGAGGCTTATGAAATGCCTTCA-3′,5′- GAGGCTTATGAAATGCCTGCC-3′, and 5′-CCCTTACCCTCTCCCACAAA-3′, which distinguishes wild type and mutant alleles, followed by sequencing verification. Chimeric mice were produced by blastocyst microinjection according to standard procedures and crossed to C57BL/6N mice for germline transmission, and homozygous mutant *SNCA* S129A mice were obtained by intercrossing heterozygous F1 mice. Mice were genotyped by PCR with primers (5′-CTCTCTGTAGGGTGAGGAGG-3′ and 5′-ACCATGATTCATAACTGCCCGTTG-3′) flanking the ssODN donor template, and wild-type, heterozygous, and homozygous animals were identified by sequencing the resulting PCR fragments with a nested primer 5′-TATCCCTTACCCTCTCCCAC-3′. All mice were maintained in a mixed 129S6 and C57BL/6N background, and age- and sex-matched wild-type and homozygous mice were used for experiments.

### Plasmids and lentiviral production

Rat αS WT or rat αS S129A synthetic cDNA sequences were digested by SpeI/NotI restriction enzymes and ligated into the respective sites of pLVX-EF1a-IRES-ZsGreen1 (TaKaRa), which drives transgene expression by the EF1a promoter. Lentiviral packaging was carried out in 293-T cells as described^34^. Briefly, 293-T cells were transfected with αS WT or S129A plasmids along with pMD2.G and psPAX2 (packaging plasmids: Addgene #12259 and #12260, respectively). Culture supernatant containing viral particles was further purified/concentrated by ultracentrifugation at 100,000 × *g*. The viral pellet was then resuspended in neurobasal medium supplemented with B-27 and Glutamax (Gibco). On an average we obtained about 2.5 × 10^6^ viral particles per µL.

### Chemicals

Picrotoxin (Sigma #P1675), bicuculline (Sigma #505875), tetrodotoxin (Cayman #14964), BI2536 (Selleckchem #S1109), Calyculin A (Abcam #ab141784), DL-AP5 (Tocris #0105), CNQX (Tocris #0190), FK506 (Tocris #3631), digitonin (Sigma #D141), nimodipine (Sigma #482200), conotoxin (Sigma #343781-M), agatoxin (Abcam #ab120210). All combined treatment paradigms throughout the manuscript were carried out in the presence of PTX (unless otherwise stated). As controls, pharmacological inhibitor treatments were also carried out in the absence of PTX in the same experiment.

### Primary neuron culture

Primary rat cortical neurons were cultured from E18 Sprague-Dawley rats (Charles River, Wilmington, MA). Pregnant rats were euthanized with CO_2_ followed by bilateral thoracotomy. Embryonic cortices were isolated and dissociated with trypsin/EDTA and trituration. 250,000 cells were plated on poly-D-lysine coated 24-well plates and cultured in neurobasal medium supplemented with B-27 and GlutaMAX (Gibco). Half of the medium was replaced every 4 days. For hippocampal neuron culture, hippocampi were isolated either from P1 Sprague-Dawley rat pups (Charles River, Wilmington, MA) or *SNCA*^*−/−*^ rat pups (ENVIGO, St. Louis, MO) and dissociated using the papain dissociation system from Worthington (Lakewood, NJ). The *SNCA*^*−/−*^ rat background is Sprague-Dawley. This strain (HsdSage:SD-*SNCA*^em1Sage^) developed by SAGE Labs, Inc, St. Louis, MO in collaboration with The Michael J. Fox Foundation contains a deletion of the endogenous *SNCA* gene, generated by CRISPR/Cas9 targeting. For patch clamp recordings, hippocampal neurons were plated on glass coverslips coated with poly-D-lysine and laminin in neurobasal medium supplemented with B-27 and Glutamax. BrainPhys neuronal medium supplemented with SM1 (Stemcell Technologies) was used for subsequent media changes. DIV5 neurons were transduced with αS WT or αS S129A lentivirus at MOI 5.

### Sequential protein extraction

Isolation of cytosolic and membrane protein fractions was carried out as described^34^. Briefly, the on-plate sequential extraction was performed in 24-well plates. Neurons were rinsed once with HBSS. In total, 125 µL of buffer “cytosol” (10 mM PIPES pH 7.4, 100 mM NaCl, 300 mM sucrose, 5 mM MgCl_2_, 5 mM EGTA and 900 mg/mL digitonin) were added per well. The plates were incubated at 37 °C for 15 min. The resultant cytosolic protein fractions were collected into 1.5 mL tubes. Subsequently, 125 µL buffer “membrane” (10 mM PIPES pH 7.4, 100 mM NaCl, 300 mM sucrose, 5 mM MgCl_2_, 5 mM EGTA, 0.5% Triton X-100 and protease inhibitors) were added per well followed by incubation at 37 °C for 15 min. The resultant membrane fractions were collected into 1.5 mL tubes.

### Synaptosome preparation

Synaptosomes were purified from DIV17-21 rat cortical neurons as described^43^, with modifications. Briefly, 20 million cells per condition (DMSO or PTX) were harvested in HBSS. Cell pellets were homogenized in 10% (w/v) of homogenization buffer (0.32 M sucrose, 4 mM HEPES, 20 mM DTT, 5 mM EDTA, protease inhibitors) with slow and uniform strokes at ~600 rpm in a Potter-Elvehjem homogenizer. The homogenate was then centrifuged at 14,500 × *g* for 10 min. The pellet was resuspended in homogenization buffer and loaded on top of a three-step (3, 10, and 23%) Percoll gradient (Sigma, Natick, MA). The mixture was centrifuged at 18,700 × *g* for 10 min and the synaptosome-rich interface between 10 and 23% Percoll was carefully recovered using a glass Pasteur pipette. To remove the Percoll from the isolated fraction, the sample was resuspended in saline buffer (0.32 M sucrose, 140 mM NaCl, 5 mM KCl, 5 mM NaHCO_3_, 1.2 mM NaH_2_PO_4_, 1 mM MgCl_2_, 20 mM HEPES pH 7.4) and centrifuges at 19,000 × *g* for 12 min. The synaptosomal pellet was resuspended in saline buffer to obtain a final concentration of ~1 mg/mL.

### Western blots

Samples for electrophoresis were prepared by lysing cells in lysis buffer (10 mM PIPES pH 7.4, 100 mM NaCl, 300 mM sucrose, 5 mM MgCl_2_, 5 mM EGTA, 0.5% Triton X-100), the addition of 4X Laemmli buffer supplemented with 1.25% β-mercaptoethanol, and boiling for 5 min. Samples were electrophoresed on NuPAGE 4–12% Bis-Tris gels with NuPAGE MES-SDS running buffer and SeeBlue Plus2 molecular weight marker (all by Invitrogen) at 140 V and transferred in the iBlot 2 system (Invitrogen) to nitrocellulose membranes (iBlot 2 NC regular stacks; IB23001). Membranes were fixed for 10 min in 0.4% paraformaldehyde (in PBS). Membranes were then blocked in blocking buffer (5% milk in TBST) for 1 h and incubated in primary antibody in blocking buffer overnight at 4 °C. Membranes were washed 5 × 5 min in TBST. Secondary antibodies were prepared in the blocking buffer and incubated for 1 h at RT. Membranes were washed 5 × 5 min in TBST and scanned (Odyssey CLx, Li-Cor). All western blots were processed in parallel and derive from the same experiment.

### Antibodies

αS (BD Biosciences, Syn1, 610787, Cell Signaling, D37A6); αS pS129 (Abcam, EP1536Y, ab51253; Abcam, MJF-R13, ab168381; Cell Signaling, D1R1R, 23706S – EP1536Y was used throughout the study unless otherwise stated), calnexin (Sigma #C4731), αS pY39 (Biolegend, A15119B), αS pY125 (Abcam, ab131466), αS Y136 (Abcam, ab194775), GAPDH (Santa Cruz, 6C5, sc-32233 and Abcam, 6C5, ab8245), Plk2 (Abclonal #A7066), c-fos (Abcam, 2H2, ab208942). Secondary antibodies were anti-rabbit Fluorescent LiCor IRDye 800CW and anti-mouse Fluorescent LiCor IRDye 700DX. Antibodies used for immunofluorescence and in vitro kinase assays are listed below in the respective sections.

### Calcium imaging

DIV5 rat cortical neurons were infected with jRGECO1a AAV^73^ (purchased from Addgene). Calcium transients in DIV18 neurons treated with either DMSO or 20 µM PTX for 2 h were recorded using a Leica DMi8 widefield fluorescence microscope equipped with 5% CO_2_. Time series images were acquired at 250 ms/frame for 2 min at 37 °C. A customized FIJI ImageJ macro was written to analyze acquired time series data. The analysis process had two steps. (A) Identification of cells with signal change during the time series: raw data went through smoothing (Mean filter, radius 2) and stack projection (standard deviation), then auto-threshold algorithm “Triangle” was applied to create a mask image of positive cells. The “Watershed” method was used to separate touching cells and cell ROIs were selected by the “Analyze Particle” function in ImageJ. (B) For each cell ROI selected by step A, a profile of mean fluorescence intensity inside the ROI was plotted as a 1-d image, the peak positions were identified by the “Find Maxima” function, and false peaks due to noise were removed. Peak positions of each individual cell ROI selection were saved as an Excel file. Finally, the correlation of peak positions among all cell ROIs was measured (the correlation was defined as how compact of peak position clusters were). Both macros are available in a GitHub repository (https://github.com/udettmer/dettmerlab).

### Whole-cell patch clamp recording of sEPSC and sIPSC

*SNCA*^*−/−*^ rat hippocampal neurons were infected at DIV5 to express αS WT or S129A. The bicistronic system enabled the expression of untagged αS variants along with ZsGreen1, a fluorescent reporter with an excitation wavelength at 496 nm and emission at 506 nm. Neurons were identified for recording by expression of ZsGreen1. Whole-cell patch-current recordings from primary hippocampal neurons at DIV 14–18 were obtained at 32–35 °C in the external solution: 145 mM NaCl, 5 mM KCl, 10 mM HEPES, 10 mM D-glucose, 2 mM MgCl_2_, 2 mM CaCl_2_ (pH 7.3) and the internal solution: 110 mM CsMeSO_4_, 10 mM NaMeSO_4_, 10 mM EGTA, 1 mM CaCl_2_, 10 mM HEPES, 10 mM TEA, 5 mM QX-314, 5 mM MgATP, 0.5 mM Na_2_GTP (pH 7.2). Spontaneous excitatory postsynaptic currents (sEPSCs) and spontaneous inhibitory postsynaptic currents (sIPSCs) were recorded in voltage clamp mode, holding cells at −60 and 10 mV, respectively. Recordings performed with a Multiclamp 700B amplifier (Molecular Devices, San Jose, CA). Signals were filtered at 2 kHz and sampled at 10 kHz with Digidata 1440A (Molecular Devices, San Jose, CA). Access resistance (Ra) was monitored following membrane rupture and dialysis, and recordings were abandoned if Ra was >15 MΩ. A pCLAMP 10.2 (Molecular Devices, San Jose, CA) was used to for data display, acquisition and storage, and offline analysis of mEPSCs was performed using the MiniAnalysis software (synaptosoft). E/I amplitude ratio was derived from Fig. 6e and i. Respective averages in Fig. 6f (αS WT or αS S129A) were divided by respective individual values (αS WT or αS S129A) in Fig. 6j to obtain E/I amplitude ratio. Each circle represents an individual cell. The data presented include two independent cultures. Statistical analyses were performed using GraphPad Prism 9. Data were presented as mean ± SD.

### Immunofluorescence microscopy

To examine the subcellular distribution of pS129 in neurons after PTX treatment, multiple labeling immunocytochemistry was performed on fixed cells. DIV18 cortical neurons were treated with DMSO or 20 µM PTX for 2 h. Sequential fixation was performed to preserve the integrity of neurons. First, 100 µL of 4% paraformaldehyde in PBS were directly added to 50,000 neurons grown on glass coverslips in a 24 well-plate containing 1 mL culture medium. After a 5 min incubation at 37 °C, medium was carefully aspirated, and cells were fixed with 4% paraformaldehyde in PBS for 10 min at room temperature. Cells were permeabilized using 0.3% Triton-X 100 and blocked in 5% normal donkey serum, after which they were incubated overnight at 4 °C in PBS containing primary antibody dilutions. An antibody directed against pS129 (Abcam, EP1536Y, #ab51253; 1:3000) was combined with antibodies against synapsin-I/II (Synaptic Systems; #106.004; 1:400), neurofilament (BioLegend #SMI-312, 1:200) and MAP2 (Abcam #ab5392; 1:1000) for the visualization of presynaptic terminals/boutons, axons, and dendrites/somata, respectively. After washing in PBS, cells were incubated in the appropriate fluorophore-conjugated secondary antibodies (diluted 1:400 in PBS) for 1.5 h at RT. Coverslips were mounted in Vectashield Antifade Mounting Medium containing DAPI. Negative primary antibody controls were performed to confirm specificity of staining patterns under the applied imaging settings. High-resolution confocal microscopy was done using a Leica TCS SP8-STED microscope (Leica Microsystems) using a HC PL APO CS2 63x/1.40 Oil objective. All signals were detected using gated hybrid detectors in counting mode. Sections were sequentially scanned for each fluorophore by irradiation with a pulsed white light laser at different wavelengths. All images were taken at the same zoom (0.9) and resolution (4096 × 4096; pixel size = 50 nm). Focal planes were established based on autofocus on synaptophysin positive puncta. Image processing and analysis were done in FIJI ImageJ Version 2.1.0/1.53c. All steps in image processing were the same for all images included in the analysis. Integrated optical density values were determined for the pS129 signal, which was compared between PTX and DMSO (control) conditions. For colocalization analysis, thresholding was done for all images using the same built-in FIJI algorithm (IJ_IsoData) after which the Colocalization Highlighter plugin was used to identify pS129 positive puncta colocalizing with synapsin I/II. The amount of co-localization was expressed as the percentage of total synapsin positive area in the image. Statistical analysis was performed using the Graphpad Prism 9 software. After elimination of outliers (ROUT, Q = 5%), statistical comparison between PTX and DMSO conditions was done using student’s t-tests.

### In vitro kinase assay

In a total reaction volume of 30 µL, 2 µM recombinant αS (prepared as published^74^) were made up in a buffer containing 50 mM Tris HCl, 50 µM ATP, 20 mM MgCl_2_, 5 mM DTT. The reaction was initiated by the addition of 100 ng human recombinant Plk2 (Invitrogen) and incubated at 30 °C for 5 min. Then the reaction was quenched by NuPAGE sample buffer (Invitrogen) followed by 5 min heating at 95 °C. Reactions were performed in the presence or absence of 0.2 mM small unilamellar vesicles consisting of soybean L-α-Phosphatidylinositol (Sigma Aldrich). Samples were run on SDS-PAGEs alongside 2 µM human recombinant pS129 αS protein (Proteos) as a control. For Western blots, Syn1 (BD Transduction labs) and MJF-R13 (Abcam) were used to detect total and pS129 αS, respectively.

### NMR

^15^N-labeled human αS WT was expressed and purified as described previously^50^. Samples of ^15^N-labeled WT αS were solubilized in a buffer containing 50 mM Tris HCL, 50 μM ATP, 20 mM MgCl2, 5 mM DTT for a final solution concentration of ~100 µM. The phosphorylation reaction was initiated by the addition of 2 µM (final concentration) of human recombinant Plk2 (Invitrogen) and incubated at 30 ˚C for 5 min. ^1^H-^15^N HSQC spectra of ^15^N-labeled αS WT were recorded directly after the incubation period with the addition of 10% D_2_O in a 5-mm NMR tube. Spectra were recorded on a 600 MHz Bruker AVANCE II spectrometer (Bruker, Billerica, MA) equipped with a Prodigy CryoProbe at 15 ˚C. Non-uniform sampling (NUS) of 25% using Poisson Gap distribution was applied and reconstructed using the hmsIST protocol^51^. Resonances were assigned, where possible, by inspection and comparison with the previously assigned spectra of WT αS (BRMB 6968 and 5744). Data processing and analysis were performed with the Bruker TopSpin software and CcpNmr Analysis^52^. Perturbations in the chemical shift values for ^1^H and ^15^N were calculated as [(∆δ^1^H)^2^ + (0.15·∆δ^15^N)^2^]^1/2^.

### Environmental enrichment

C57BL/6NCrl inbred mice from Charles River (Wilmington, MA) were used. We followed the experimental set-up as described previously^26^. Briefly, 4-week-old mice were randomly divided into two groups: standard housing (SH) or enriched environment (EE). Each group contained 8–12 mice with both males and females. SH utilizes a normal housing cage (25/20/15 cm). EE cages were large (60/38/20 cm). EE cages contained 1 InnoDome + InnoWheel and multiple toys (such as bio-tunnel, pup tents, crawls balls, bio-hut, mouse arch etc.) and objects of varying shapes and colors. All toys were purchased from Bio-Serv NJ (Phillipsburg, NJ). The toys were changed daily. Mice were periodically monitored for their ability to explore the objects. Mice spent about 6 h every day for 8 weeks in EE cages. Each day after the training male and female mice were separated and transferred to regular cages. Mice that became pregnant were removed from the analysis. All females became pregnant halfway during the training, so they were transferred to different cages (and excluded from analysis) to avoid separating neonates from lactating females. Control SH mice were housed in the same room in standard cages.

### Preparation of hippocampal slices

One mouse each from SH and EE cohorts were euthanized by isoflurane. Brains were immediately removed and transferred into ice-cold oxygenated (95% O_2_ and 5% CO_2_) cutting buffer (206 mM sucrose, 2 mM KCl, 2 mM MgSO_4_, 1.25 mM NaH_2_PO_4_, 1 mM CaCl_2_, 1 mM MgCl_2_, 26 mM NaHCO_3_, 10 mM D-glucose (pH 7.4; 315 mOsm). Using a vibroslicer, slices of 350 µm thickness were made and transferred into a dish containing artificial cerebrospinal fluid (aCSF: 125 mM NaCl, 2 mM KCl, 2 mM MgSO_4_, 1.25 mM NaH_2_PO_4_, 2.5 mM CaCl_2_, 26 mM NaHCO_3_ and 10 mM D-glucose [pH 7.4; 310 mOsm]). The slices were allowed to recover for about 90 min in aCSF before recording.

### Field recordings

LTP recordings were performed as described previously^26,75^, with modifications. Individual slices were recorded by transferring one at a time into a recording chamber containing oxygenated aCSF. Recordings were performed at 26 °C. With appropriate stimulation protocols field excitatory postsynaptic potentials (fEPSP) were recorded in the CA1 region of the hippocampus. A bipolar stimulating electrode was placed in the Schaffer collaterals to deliver test or conditioning stimuli. A glass recording electrode filled with aCSF was placed in stratum radiatum of CA1, about 150–300 µm from the stimulating electrode. A test stimulus at 0.05 Hz was first used to record test responses 20 min prior to the experiment to ensure the stability of the response. To induce standard high-frequency stimulation of LTP, two consecutive trains (1 s) of stimuli separated by 20 s at 100 Hz were applied to slices (HFS). This protocol induced LTP for ~1.5 h. Using an alternative protocol, a weak-HFS stimulation was performed. This weak-HFS contained only one train of 100 Hz stimulation. For STP, multi-pulse stimulations were applied at 25 Hz with a 40 ms interval. The field potentials were amplified 100x by Axon Instrument 200B and digitized with Digidata 1322 A. Using pClamp, 9.2 (Molecular Devices, San Jose, CA) traces were obtained and subsequently analyzed by the Clampfit 9.2 program (Molecular Devices, San Jose, CA).

### Statistical analyses

We performed: (1) Paired and unpaired t*-*test (two-tailed) with or without Welch’s corrections (Figs. 1b, h, j–l, 2f, 2a–d, 3e, 4d, 5b, 6d, f, h, j, l, 7i, j, l, Supplementary Figs. 1c, 2a and 8a); (2) Brown-Forsythe and Welch ANOVA with Dunnett’s T3 post hoc test for multiple comparisons (Figs. 1a, c–g, 2e, f, 3a, c, d, f, g, 4c, d, Supplementary Figs. 2, 3b–d); (3) RM one-way ANOVA with post hoc Sidak’s multiple comparisons test (Fig. 3a, Supplementary Fig. 5c–e), (4) 2-way ANOVA with post hoc Tukey’s multiple comparisons test (Fig. 4b), and (5) 2-way ANOVA with Sidak’s multiple comparisons test (Supplementary Fig. 7b). We used GraphPad Prism Version 9 following the program’s guidelines for all analyses. Graphs represent means ± SD. Criteria for significance were: *P < 0.05, **P < 0.01, ***P < 0.001, and ****P < 0.0001. Sufficient experiments and replicates were analyzed to achieve statistical significance, and these judgments were based on earlier, similar work.

### Reporting summary

Further information on research design is available in the Nature Research Reporting Summary linked to this article.

## Supplementary information

Supplementary figures

Reporting Summary

Movie 1

Movie 2

Movie 3

## Data Availability

All data generated (and commercially non-available tools used for analysis) during this study are included. Non-identifying data, if found any will be made available upon request to the corresponding authors.

## Code availability

Codes used for calcium imaging analysis are available in a GitHub repository (https://github.com/udettmer/dettmerlab).
