# Supplementary material for: Dynamic physiological α-synuclein S129 phosphorylation is driven by neuronal activity
Source: NPJ Parkinsons Dis. 2023 Jan 16;9:4. doi: 10.1038/s41531-023-00444-w (PMC9842642; doi:10.1038/s41531-023-00444-w)
Supplement: Supplementary file 1 — Supplementary figures [file 41531_2023_444_MOESM1_ESM.pdf]

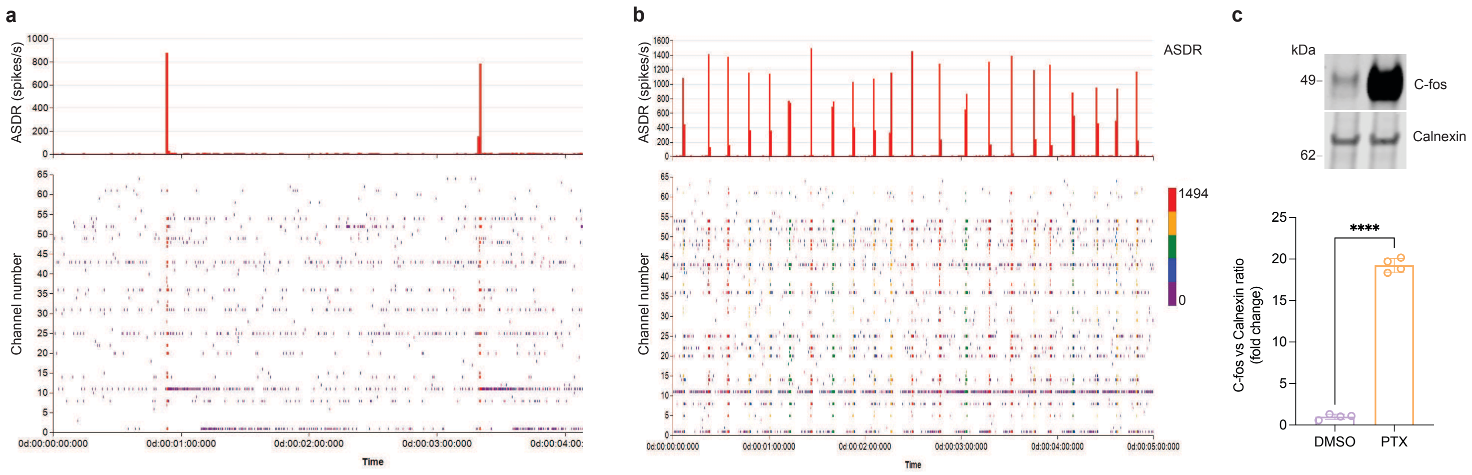

Supplementary Figure 1. PTX stimulates neuronal activity. Multiple Electrode Assay to monitor the activity of DIV18 rat cortical neurons under control conditions (**a**) and upon 20 µM PTX stimulation (**b**). Top: The array-wide spike detection rate (ASDR) across 64 electrodes in response to PTX. X-axis: electrodes from 1 to 64 (left to right); Y-axis: ASDR. Bottom: network activity from individual electrodes (64 in total) over a 4-5 min recording. X-axis and Y-axis represent time (min) and channel numbers = electrodes, respectively. **c**, 20 µM PTX stimulation of DIV18 rat cortical neurons for 2 h. WB for C-fos and calnexin (loading control). N=4 independent sample preparations. ****, $p<0.0001$. Mean +/- SD. Unpaired t-test; two-tailed.

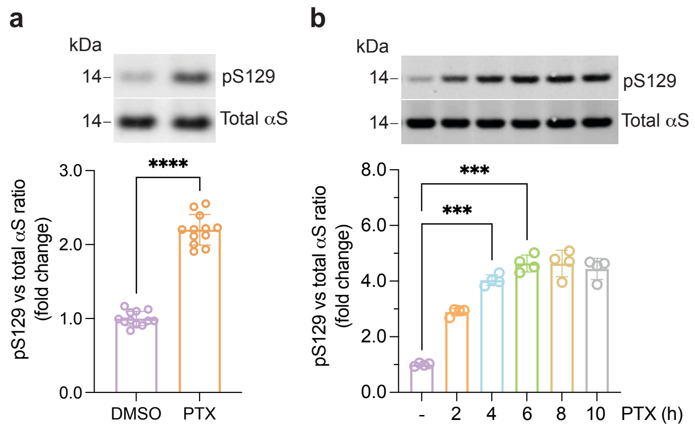

Supplementary Figure 2. Activity-dependent pS129 persists for hours and plateaus eventually. DIV17-21 cortical neurons treated with 20 µM PTX. **a**, Neurons treated with PTX 24h. WB for total αS and pS129 (D1R1R). N=3 independent experiments on different days, n=12 biological replicates total; p<0.0001. Mean +/- SD. Unpaired t-test; two-tailed. **b**, Neurons treated with PTX for durations as indicated. WB for total αS and pS129. N=4 independent sample preparations. ***, p<0.0003; Mean +/- SD. Brown-Forsythe and Welch ANOVA with *post hoc* Dunnett's T3 multiple comparisons test.

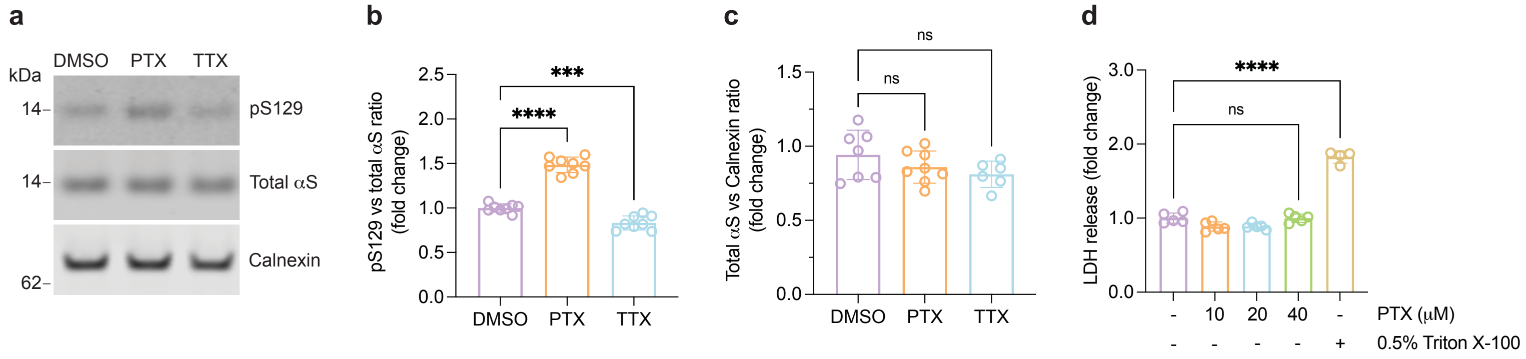

Supplementary Figure 3. 24 h PTX stimulation does alter total αS levels; brief PTX stimulation is not toxic to neurons. DIV17-21 rat cortical neurons treated with 20 μM PTX or 1 μM TTX for 24 h. **a**, WB for total αS, pS129 and calnexin. **b**, Quantification of pS129 vs total αS. **c**, Quantification of total αS vs. Calnexin (loading control). N=8 independent sample preparations. ns, not significant; ***, $p<0.0006$; ****, $p<0.0001$; Mean +/- SD. Brown-Forsythe and Welch ANOVA with Dunnett's T3 *post hoc* test for multiple comparisons. **d**, DIV17-21 rat cortical neurons treated with indicated concentration of PTX for 2h followed by LDH release assay; ns, not significant; ****, $p<0.0001$; Mean +/- SD. Brown-Forsythe and Welch ANOVA with Dunnett's T3 *post hoc* test for multiple comparisons.

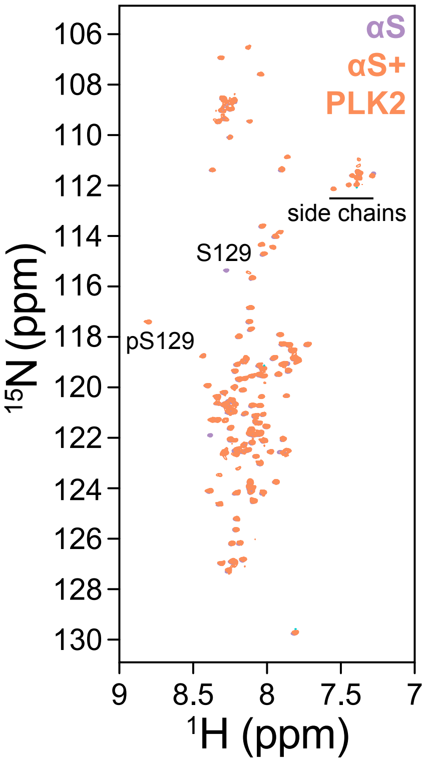

Supplementary Figure 4. NMR spectrum of recombinant human αS in the presence or absence of recombinant Plk2. This is related to Fig. 3b. 2D ^{1}H-^{15}N HSQC NMR spectrum of recombinant human αS (about 100 μM) in the presence (orange) or absence (purple) of 2 μM recombinant Plk2.

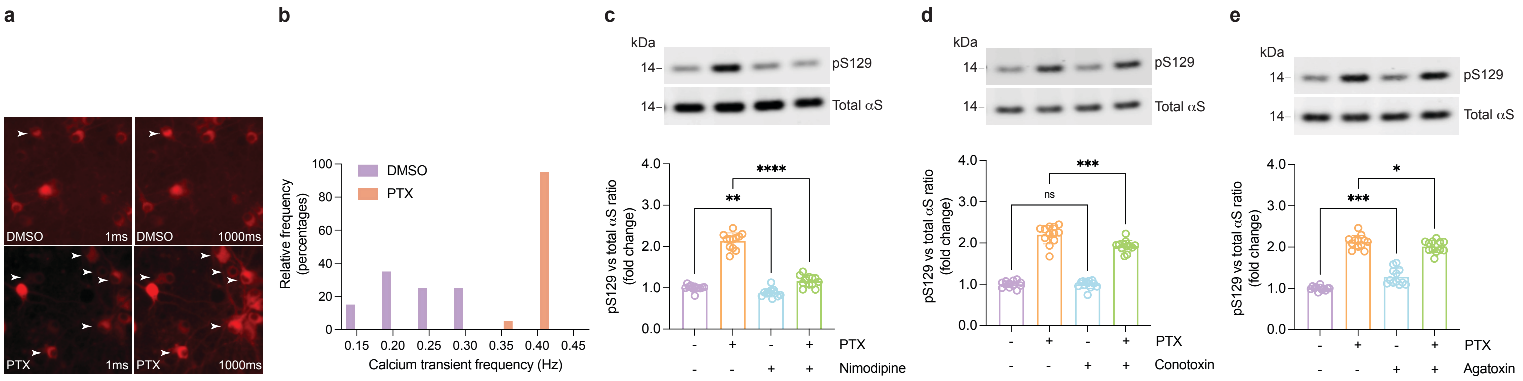

Supplementary Figure 5. Blocking of calcium channels differentially inhibits activity-dependent pS129. a, Representative images of calcium transients of neurons transduced with jRGECO1a AAV in DIV18-20 rat cortical neurons. Arrowheads represent cells undergoing calcium transients in a presented frame. **b**, Relative frequency distribution of calcium transients. N=95 individual events for DMSO, N=74 individual events for PTX. **c**, DIV17-21 rat cortical neurons treated with 20 μM PTX, 2 μM nimodipine (L-type calcium channel blocker) or PTX+nimodipine for 2 h. WB for total αS and pS129. N=3 independent experiments on different days, n=12 biological replicates total; **p<0.001; ****, p<0.0001. Mean +/- SD. RM one-way ANOVA with *post hoc* Sidak's multiple comparison test. **d**, DIV17-21 rat cortical neurons treated with 20 μM PTX, 1 μM conotoxin (N-type calcium channel blocker) or PTX+conotoxin for 2 h. WB for total αS and pS129. N=3 independent experiments on different days, n=12 biological replicates total; ns, not significant; ***, p<0.0001. Mean +/- SD. RM one-way ANOVA with *post hoc* Sidak's multiple comparison test. **e**, DIV17-21 rat cortical neurons treated with 20 μM PTX, 40 nM agatoxin (P/Q-type calcium channel blocker) or PTX+agatoxin for 2 h. WB for total αS and pS129. N=3 independent experiments on different days, n=12 biological replicates total; *, p<0.05; ***, p<0.0005. Mean +/- SD. RM one-way ANOVA with *post hoc* Sidak's multiple comparison test.

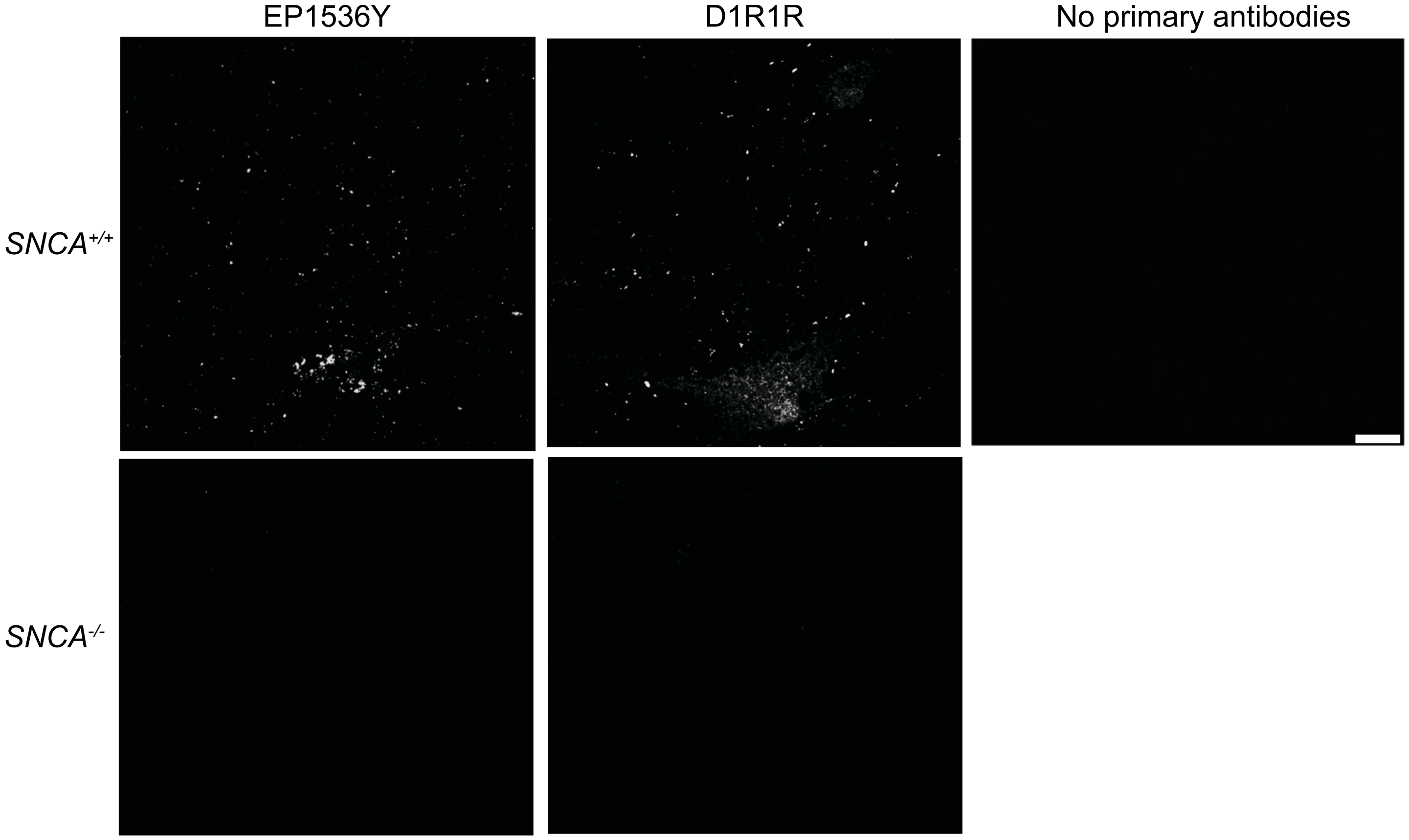

Supplementary Figure 6. Validation of presynaptic pS129 distribution. DIV18-21 cortical neurons from WT (*SNCA*$^{+/+}$) or *SNCA*$^{-/-}$ rats were subjected to immunofluorescence using EP1536Y and D1R1R, two different pS129-specific antibodies. All visualized images represent maximized 3D projection of 0.9 µm thick z-stacks. Scale bar, 10 µm.

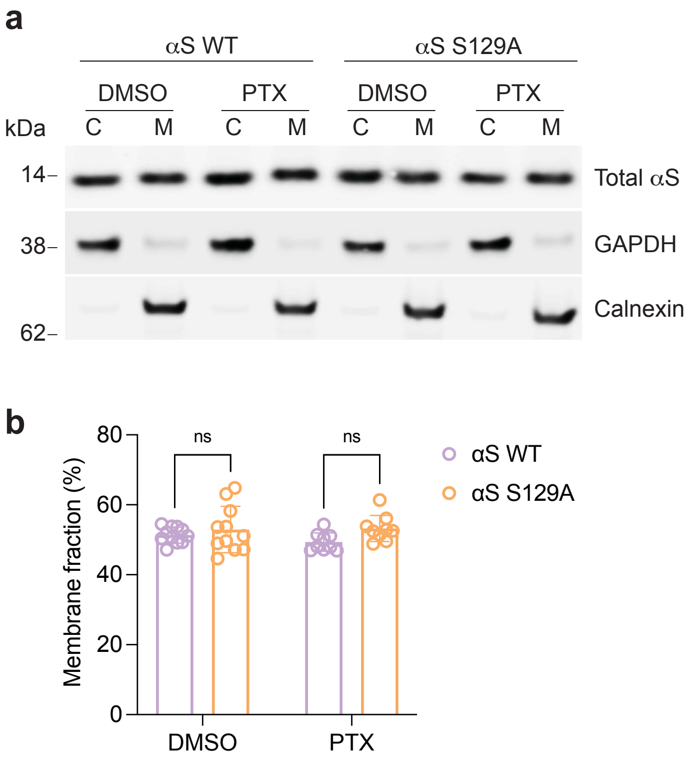

Supplementary Figure 7. pS129 does not affect the solubility of αS under both basal and stimulated conditions. a, DIV18-21 rat cortical neurons from *SNCA*$^{-/-}$ re-expressing WT αS or phospho-deficient S129A αS by lentiviral transduction. Neurons treated with either DMSO or 20 µM PTX for 2h and subjected to sequential extraction to generate cytosol (C) vs. membrane (M) protein lysates. WB for total αS, pS129, GAPDH and calnexin. **b**, Quantification of total αS in membrane fractions. N=3 independent experiments on different days, n=11 biological replicates total. ns, not significant. Mean +/- SD. 2-way ANOVA with *post hoc* Sidak's multiple comparisons test.

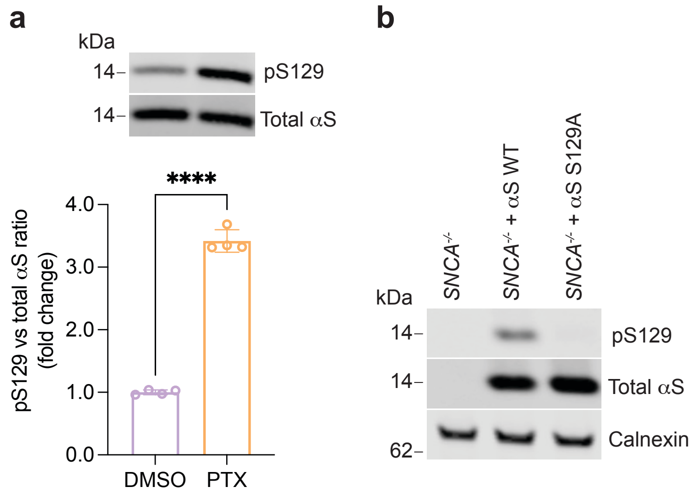

Supplementary Figure 8. Activity-dependent pS129 in rat hippocampal neurons. a, DIV18 rat hippocampal neurons treated with DMSO or 20 μM PTX for 2h. WB to total αS and pS129. N=4 independent sample preparations. ****, $p<0.0001$; Mean +/- SD. Unpaired t-test; two-tailed. **b**, DIV18 rat hippocampal neurons from *SNCA*$^{-/-}$ re-expressing αS WT or phospho-deficient αS S129A by lentiviral transduction. WB for total αS, pS129 and calnexin.

Supplementary Movie 1. A time-lapse video of mice training in an enriched environment cage (related to Fig. 1i-k).

Supplementary Movie 2. Calcium transients in control neurons (related to Fig. 1h and Supplementary Figure 5a,b).

Supplementary Movie 3. Calcium transients in PTX-treated neurons (related to Fig. 1h and Supplementary Figure 5a,b).

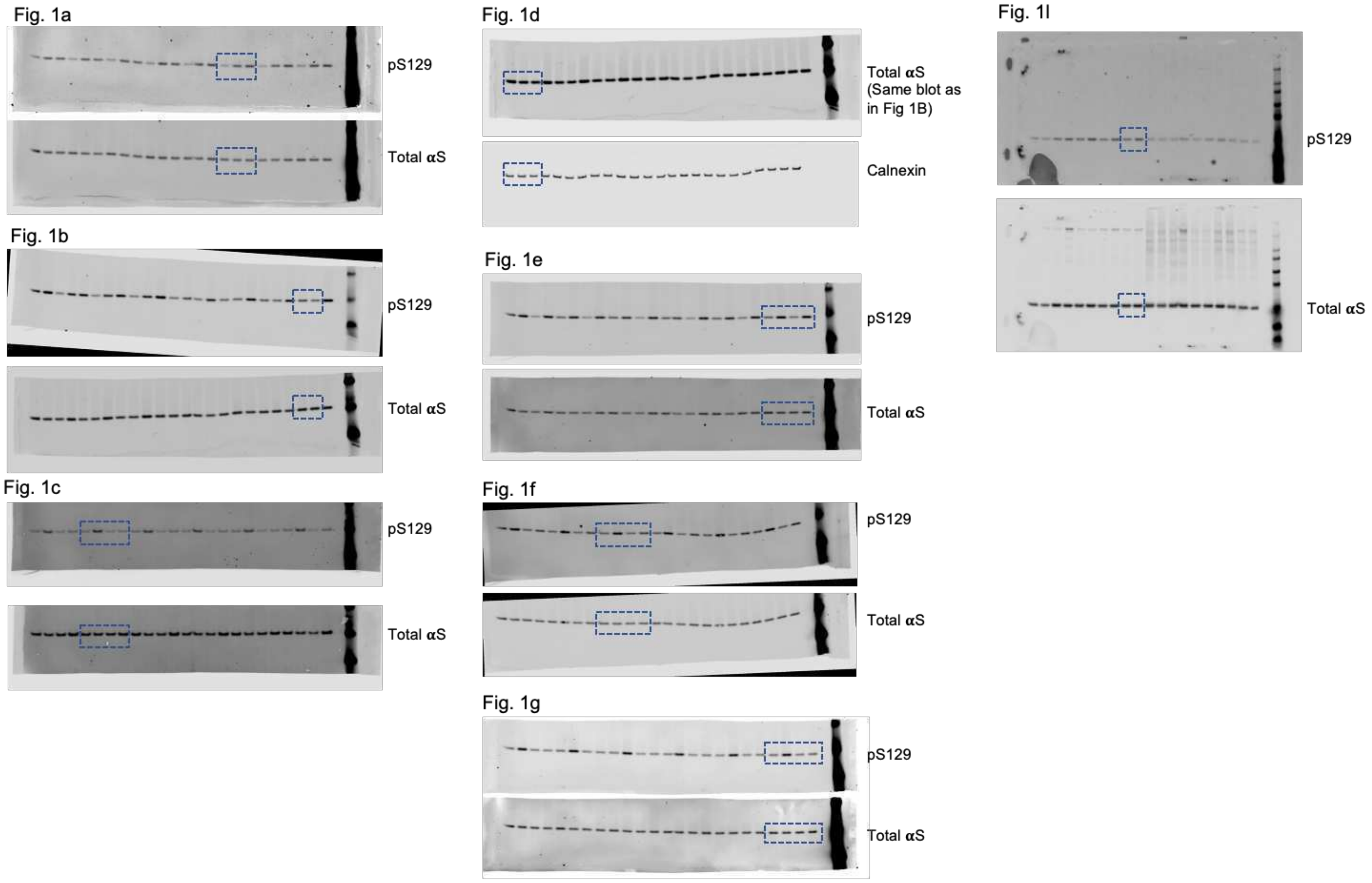

Uncropped western blot images for respective panels in Fig. 1. Cropped lanes are marked by dotted lines.

Fig. 2a

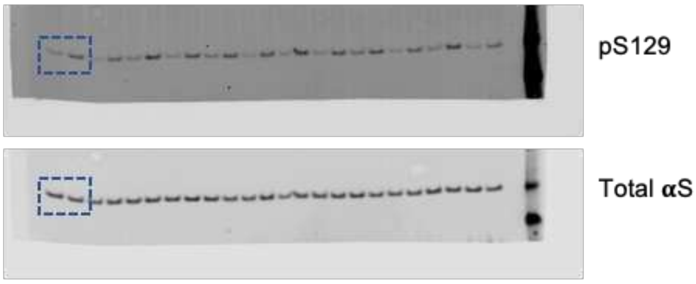

Fig. 2b

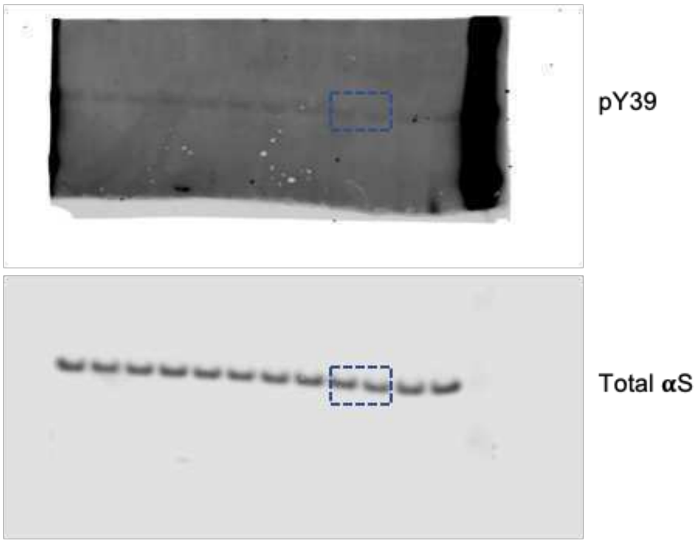

Fig. 2c

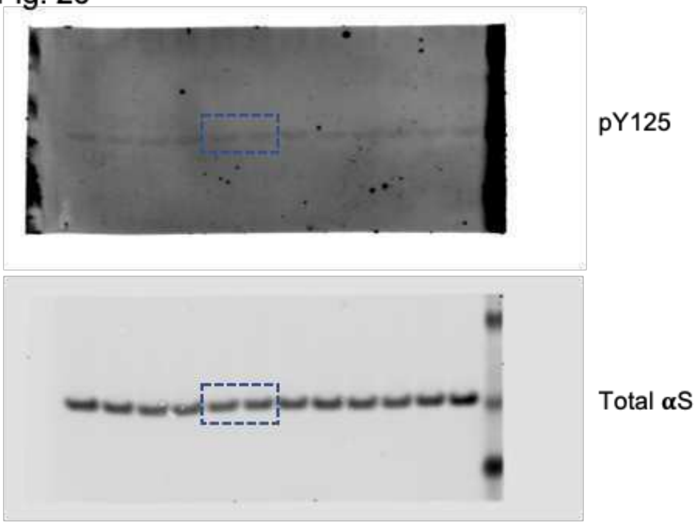

Fig. 2d

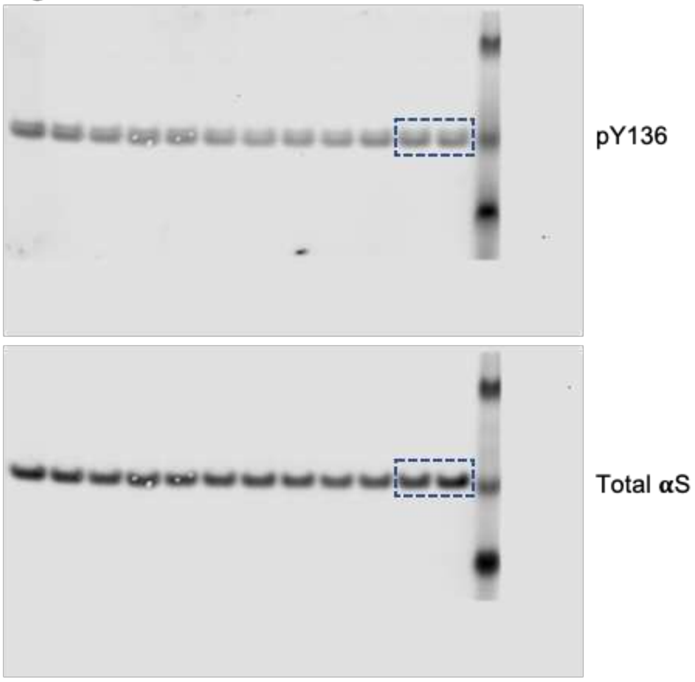

Fig. 2e

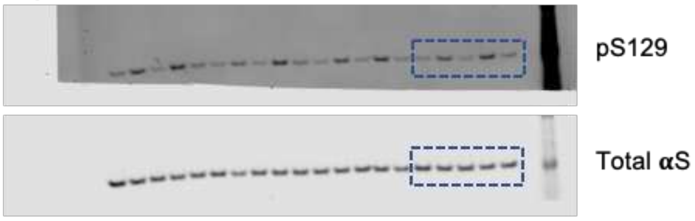

Fig. 2f

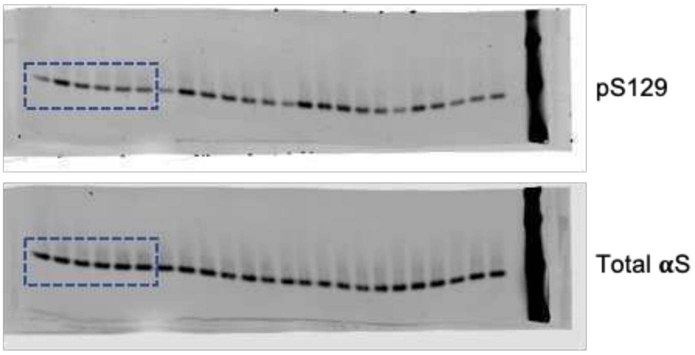

Uncropped western blot images for respective panels in Fig. 2. Cropped lanes are marked by dotted lines.

Fig. 3a

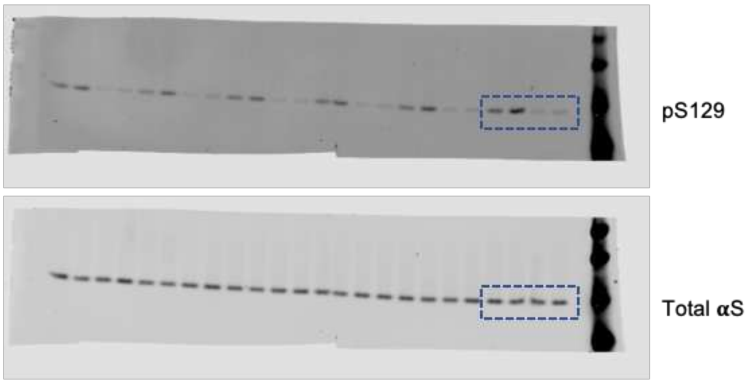

Fig. 3c

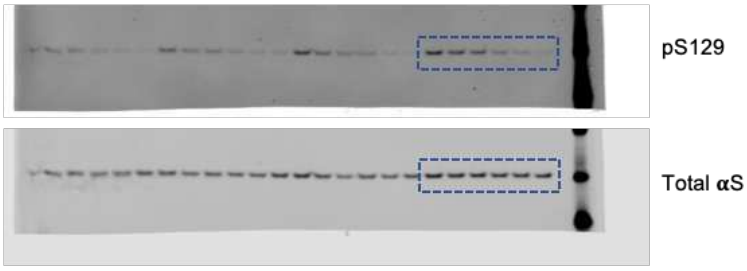

Fig. 3d

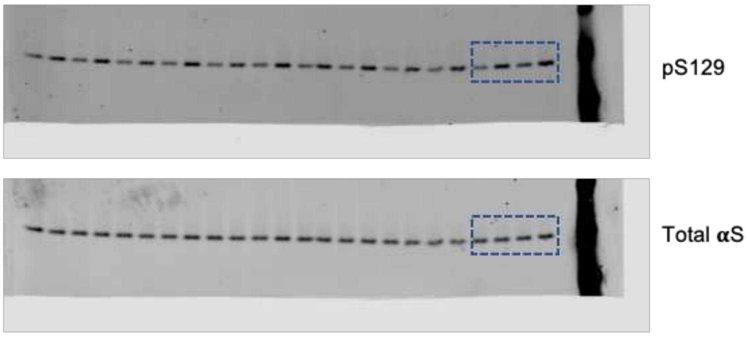

Fig. 3e

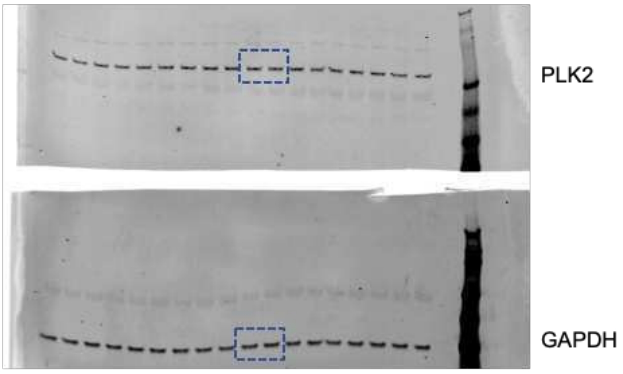

Fig. 3f

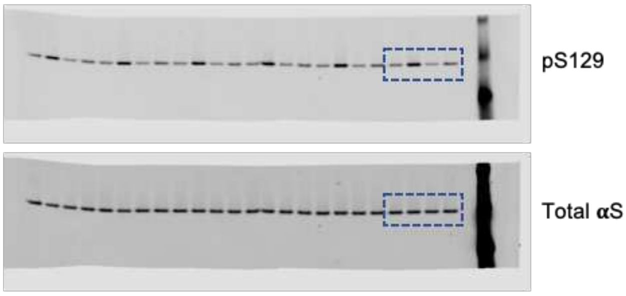

Fig. 3g

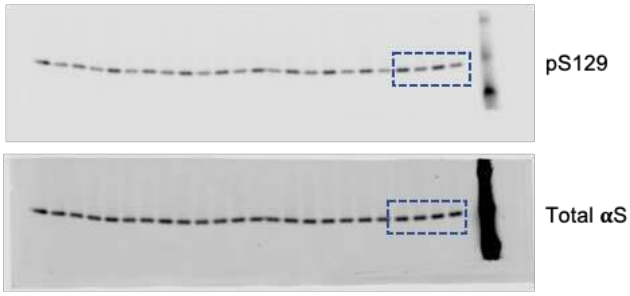

Uncropped western blot images for respective panels in Fig. 3. Cropped lanes are marked by dotted lines.

Fig. 4a

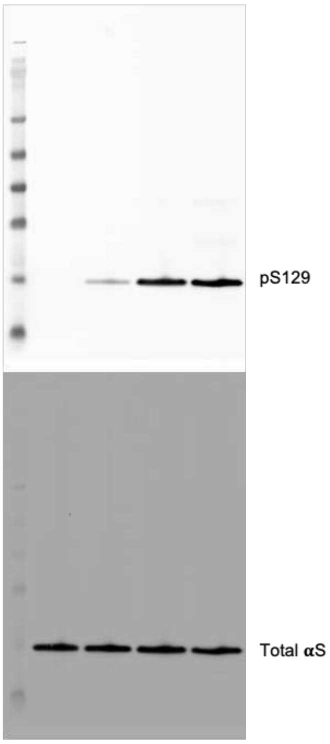

Fig. 4b

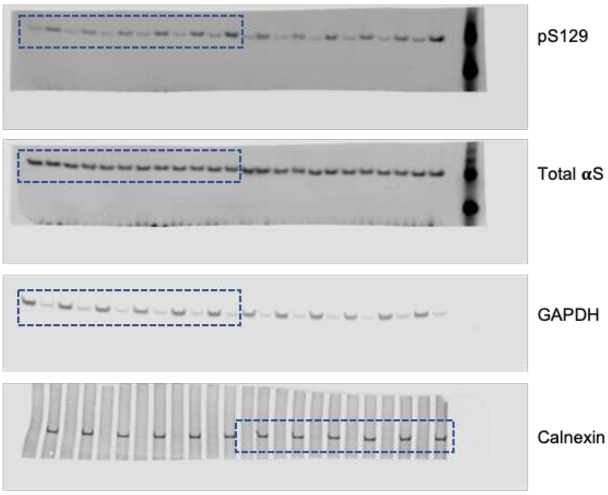

Fig. 4e

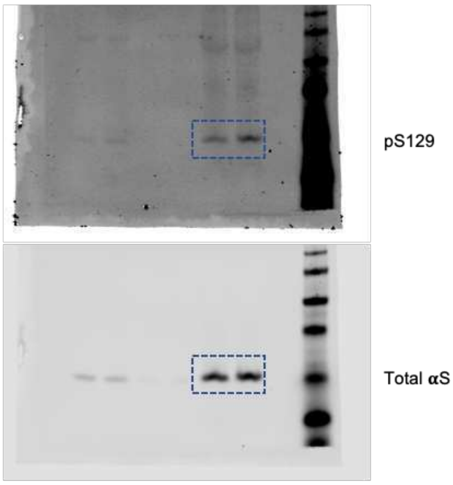

Uncropped western blot images for respective panels in Fig. 4. Cropped lanes are marked by dotted lines.

Fig. 7g

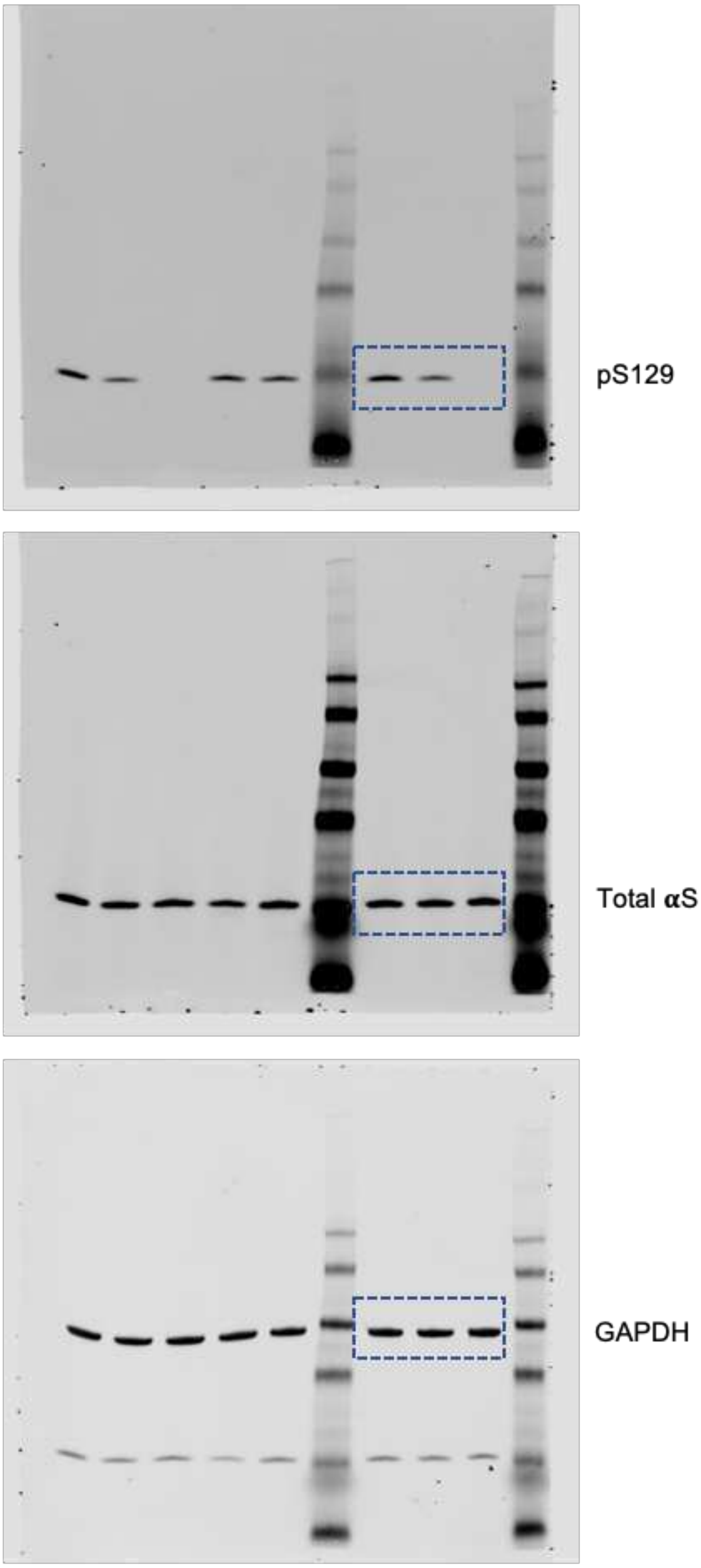

Uncropped western blot images for Fig. 7j. Cropped lanes are marked by dotted lines.

Supplementary Figure 1a

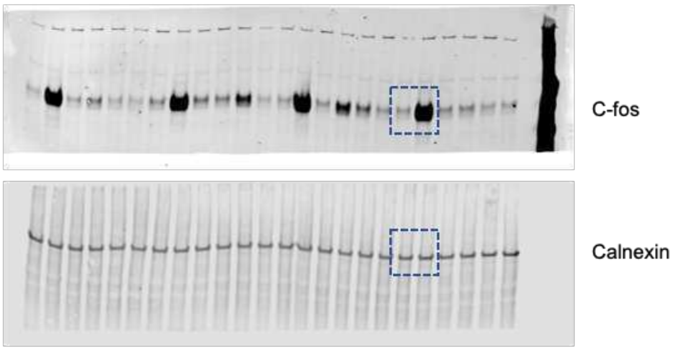

Supplementary Figure 3a

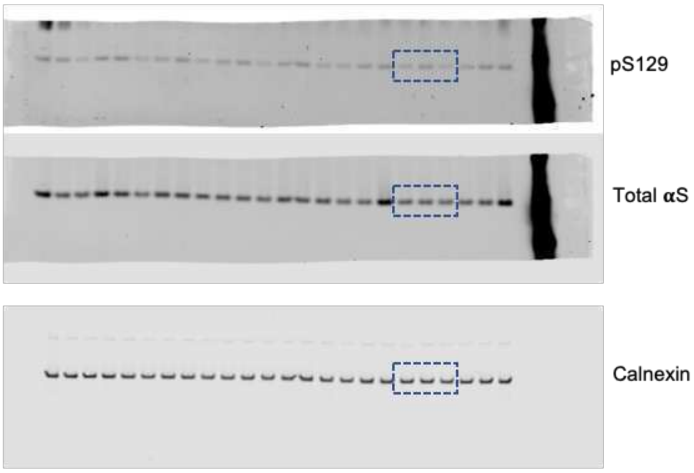

Supplementary Figure 2a

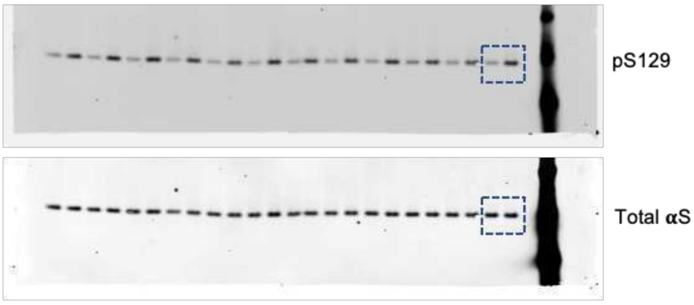

Supplementary Figure 2b

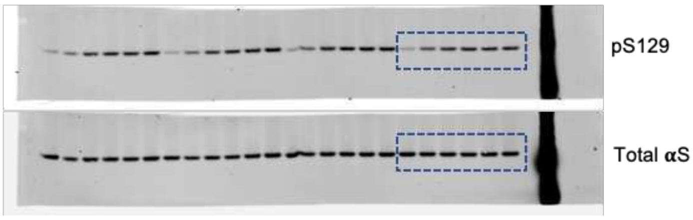

Uncropped western blot images for respective panels in Supplementary Figures 1, 2, and 3. Cropped lanes are marked by dotted lines.

Supplementary Figure 5c

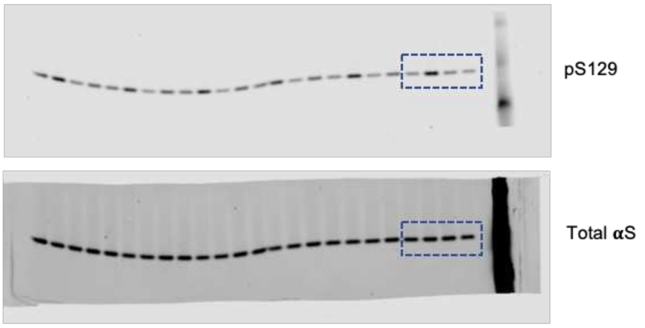

Supplementary Figure 5d

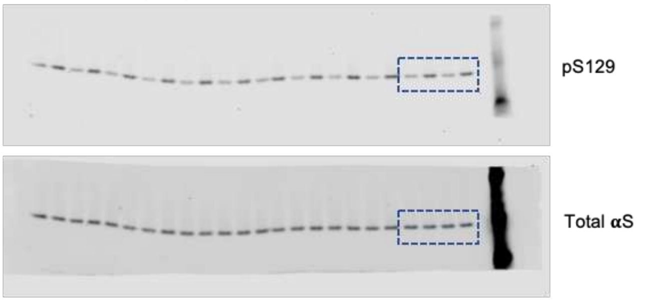

Supplementary Figure 5e

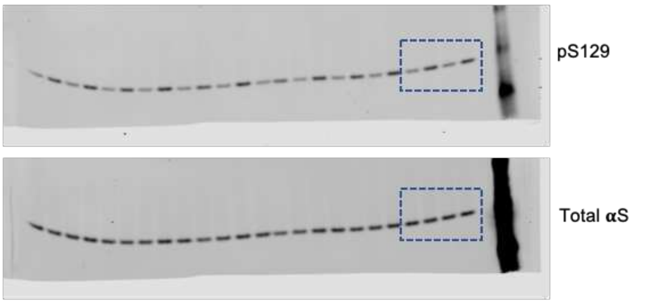

Supplementary Figure 7a

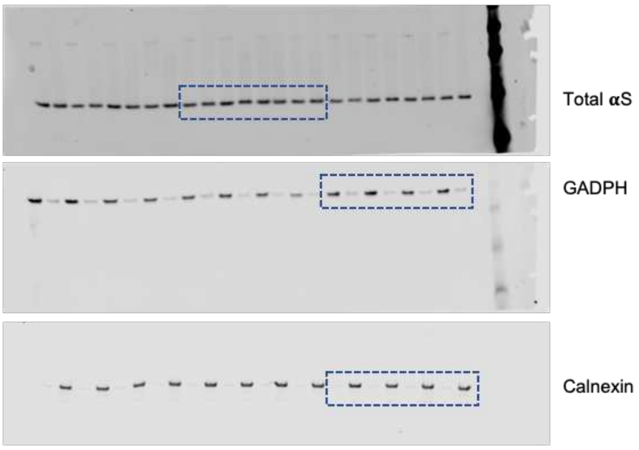

Uncropped western blot images for respective panels in Supplementary Figures 5 and 7. Cropped lanes are marked by dotted lines.

Supplementary Figure 8a

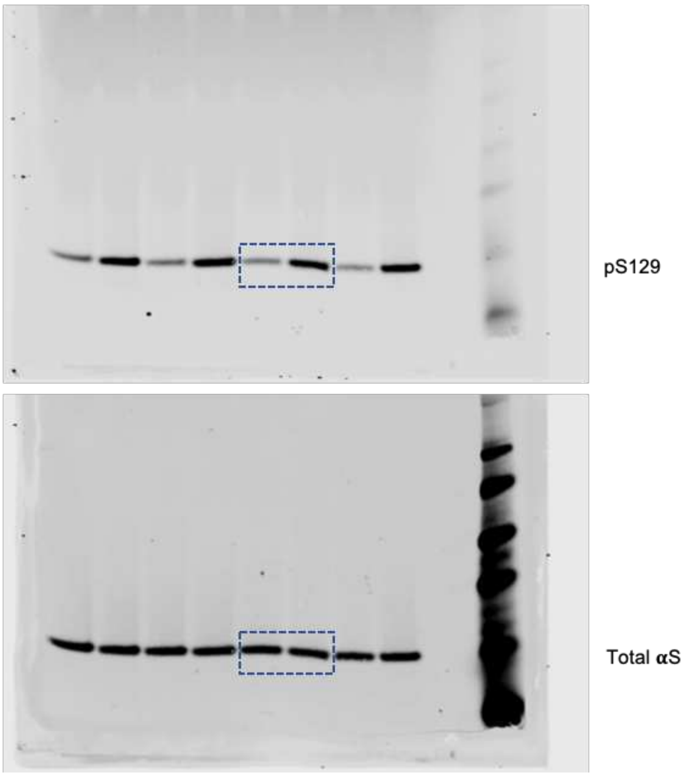

Supplementary Figure 8b

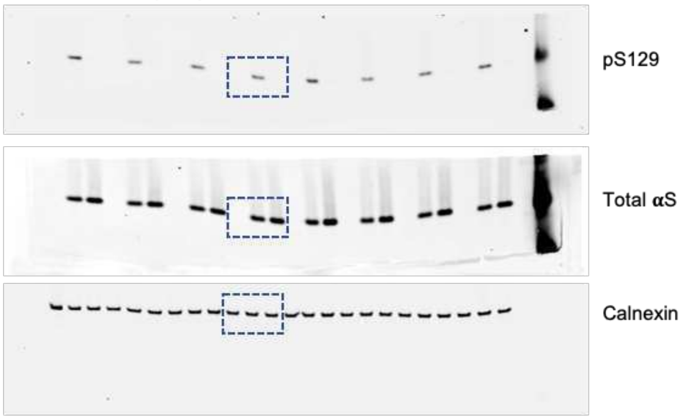

Uncropped western blot images for respective panels in Supplementary Figure 8. Cropped lanes are marked by dotted lines.
